# Classifying COVID-19 hospitalizations in epidemiology cohort studies: The C4R study

**DOI:** 10.1371/journal.pone.0316198

**Published:** 2025-02-10

**Authors:** Elizabeth C. Oelsner, Akshaya Krishnaswamy, Rafail Rustamov, Pallavi P. Balte, Tauqeer Ali, Norrina B. Allen, Howard F. Andrews, Pramod Anugu, Alexander Arynchyn, Lori A. Bateman, Jianwen Cai, Harry Chang, Lucas Chen, Mitchell S. V. Elkind, James S. Floyd, Kelley Pettee Gabriel, Sina A. Gharib, Jose D. Gutierrez, Karen Hinckley Stukovsky, Virginia J. Howard, Carmen R. Isasi, Lauren Jager, Ling Jin, Suzanne E. Judd, Alka M. Kanaya, Namratha R. Kandula, Maureen R. Kelly, Sadiya S. Khan, Anna Kucharska-Newton, Joyce S. Lee, Emily B. Levitan, Cora E. Lewis, Barry J. Make, Kimberly Malloy, Jennifer J. Manly, David Mauger, Yuan-I Min, Joanne M. Murabito, Charles G. Murphy, Arnita F. Norwood, George T. O’Connor, Victor E. Ortega, Ashmi A. Patel, Amber Pirzada, Elizabeth A. Regan, Kimberly B. Ring, Wayne D. Rosamond, David A. Schwartz, James M. Shikany, Daniela Sotres-Alvarez, Cheryl Tarlton, Janis Tse, Elman M. Urbina Meneses, Maya Vankineni, Sally E. Wenzel, Prescott G. Woodruff, Vanessa Xanthakis, Ji Hyun Yang, Neil A. Zakai, Ying Zhang, Wendy S. Post

**Affiliations:** 1 Division of General Medicine, Department of Medicine, Columbia University Irving Medical Center, New York, New York, United States of America; 2 Department of Medicine, Nassau University Medical Center, East Meadow, New York, United States of America; 3 Center for American Indian Health Research, Department of Biostatistics and Epidemiology, Hudson College of Public Health, University of Oklahoma Health Sciences Center, Oklahoma City, Oklahoma, United States of America; 4 Center for Epidemiology and Population Health, Department of Preventive Medicine, Northwestern University, Chicago, Illinois, United States of America; 5 Data Coordinating Center and Biostatistics Department, Mailman School of Public Health, Columbia University Irving Medical Center, New York, New York, United States of America; 6 Department of Medicine University of Mississippi Medical Center, Jackson, Mississippi, United States of America; 7 Division of General Internal Medicine and Population Science, Heersink School of Medicine, University of Alabama at Birmingham, Birmingham, Alabama, United States of America; 8 Collaborative Studies Coordinating Center, Department of Biostatistics, Gillings School of Global Public Health, University of North Carolina at Chapel Hill, Chapel Hill, North Carolina, United States of America; 9 NHLBI’s Framingham Heart Study, Framingham, Massachusetts, United States of America; 10 Department of Neurology, Vagelos College of Physicians and Surgeons, Columbia University, New York, New York, United States of America; 11 Department of Epidemiology, Mailman School of Public Health, Columbia University, New York, New York, United States of America; 12 Department of Epidemiology, School of Public Health, University of Washington, Seattle, Washington, United States of America; 13 Division of General Medicine, Department of Medicine, University of Washington, Seattle, Washington, United States of America; 14 Department of Epidemiology, University of Alabama at Birmingham, Birmingham, Alabama, United States of America; 15 Division of Pulmonary, Critical Care and Sleep Medicine, University of Washington, Seattle, Washington, United States of America; 16 Department of Biostatistics, School of Public Health, University of Washington, Seattle, Washington, United States of America; 17 Department of Epidemiology and Population Health, Albert Einstein College of Medicine, Bronx, New York, United States of America; 18 Department of Biostatistics, University of Alabama at Birmingham, Birmingham, Alabama, United States of America; 19 Department of Medicine, University of California San Francisco, San Francisco, California, United States of America; 20 Division of General Internal Medicine, Department of Medicine, Northwestern University, Chicago, Illinois, United States of America; 21 Division of Cardiology, Department of Medicine, Northwestern University, Chicago, Illinois, United States of America; 22 Department of Epidemiology, Gillings School of Global Public Health, University of North Carolina at Chapel Hill, Chapel Hill, North Carolina, United States of America; 23 Division of Pulmonary Sciences and Critical Care, Department of Medicine, University of Colorado Anschutz Medical Campus, Aurora, Colorado, United States of America; 24 Division of Pulmonary, Critical Care & Sleep Medicine, Department of Medicine, National Jewish Health, Denver, Colorado, United States of America; 25 Division of Biostatistics and Bioinformatics, Department of Public Health Sciences, Pennsylvania State University, State College, Pennsylvania, United States of America; 26 Department of Medicine, Boston University Chobanian and Avedisian School of Medicine, Boston, Massachusetts, United States of America; 27 Division of Pulmonary Allergy, and Critical Care Medicine, Department of Medicine, Columbia University Medical Center, New York, New York, United States of America; 28 Division of Pulmonary, Critical Care, Allergy, and Immunologic Diseases, Department of Medicine, Mayo Clinic Scottsdale, Scottsdale, Arizona, United States of America; 29 Institute for Minority Health Research, University of Illinois Chicago, Chicago, Illinois, United States of America; 30 Division of Rheumatology, Department of Medicine, National Jewish Health, Denver, Colorado, United States of America; 31 Division of Pulmonary and Critical Care, Graduate School of Medicine, University of Tennessee, Knoxville, Tennessee, United States of America; 32 Department of Environmental and Occupational Health, School of Public Heath, University of Pittsburgh, Pittsburgh, Pennsylvania, United States of America; 33 Division of Pulmonary, Critical Care, Allergy, and Sleep Medicine, Department of Medicine, University of California San Francisco, San Francisco, California, United States of America; 34 Department of Medicine, Boston University Chobanian and Avedisian School of Medicine, and Department of Biostatistics, School of Public Health, Boston University, Boston, Massachusetts, United States of America; 35 Division of Cardiovascular Medicine, Department of Medicine, Lahey Hospital and Medical Center, Burlington, Massachusetts, United States of America; 36 Department of Medicine, Larner College of Medicine at the University of Vermont, Burlington, Vermont, United States of America; 37 Department of Pathology & Laboratory Medicine, Larner College of Medicine, University of Vermont, Burlington, Vermont, United States of America; 38 Division of Cardiology, Department of Medicine, Johns Hopkins University, Baltimore, Maryland, United States of America; UN Mehta Institute of Cardiology and Research Center, INDIA

## Abstract

**Rationale:**

Robust COVID-19 outcomes classification is important for ongoing epidemiology research on acute and post-acute COVID-19 conditions. Protocolized medical record review is an established method to validate endpoints for clinical trials and cardiovascular epidemiology cohorts; however, a protocol to adjudicate hospitalizations for COVID-19 among epidemiology cohorts was lacking.

**Objectives:**

We developed a protocol to ascertain and adjudicate hospitalized COVID-19 across a meta-cohort of 14 US prospective cohort studies. This report describes the first three years of protocol implementation (October 1, 2020—October 1, 2023) and evaluates its repeatability and performance compared to classification by administrative codes.

**Methods:**

The protocol was adapted from cohort approaches to clinical cardiovascular events ascertainment and adjudication. Potential COVID-19 hospitalizations and deaths were identified by self-/proxy-report and, in some cases, active surveillance. Medical records were requested from hospitals and adjudicated for COVID-19 outcomes by clinically trained personnel according to a standardized rubric. Inter-rater agreement was assessed. The sensitivity and specificity of discharge diagnosis codes was compared to adjudicated diagnoses.

**Measurements and main results:**

The study obtained medical records for 1,167 potential COVID-19 hospitalizations, which underwent protocolized adjudication. Adjudication confirmed COVID-19 infection was present for 1,030 (88%) events, of which COVID-19 was not the cause of hospitalization for 78 (8%). Of 952 hospitalizations determined by adjudicators to be caused by COVID-19, 319 (34%) participants were critically ill and 210 (22%) died. Pneumonia was confirmed in 822 (86%) and acute kidney injury in 350 (37%); other cardiovascular and thrombotic complications were rare (2–5%). Interrater reliability among adjudicators was high (kappa = 0.85–1.00) except for myocardial infarction (kappa = 0.60). Compared to adjudication, sensitivity of discharge diagnosis codes was higher for pneumonia (84%) and pulmonary embolism (81%) than for other complications (48–70%).

**Conclusions:**

Protocolized adjudication confirmed four out of five COVID-19 hospitalizations in a US meta-cohort and confirmed cases of pneumonia, pulmonary embolism, and other conditions that were not indicated by discharge diagnosis codes. These results highlight the importance of validating health outcomes for use in research on COVID-19 and post-COVID-19 conditions, and some limitations of claims-based data.

## Introduction

Coronavirus disease 2019 (COVID-19) has been a leading cause of hospitalizations and deaths since 2020 [1, 2]. Large population-based studies with accurate characterization of hospitalized COVID-19 and its acute complications–as well as clinical, biomarker, and lifestyle data from before and after infection–are urgently needed to understand mechanisms of acute disease and post-acute sequelae of COVID-19 (PASC), which impacts up to 57% of hospitalized COVID-19 patients [3].

Many extant US cohort studies have collected extensive data on clinical and subclinical diseases and their risk factors, including behavior, cognition, biomarkers, and social determinants of health. Since enrollment for these studies was completed prior to the COVID-19 pandemic, they offer a unique opportunity to study risk factors for incident COVID-19 while minimizing the referral, survival, and recall biases that are common to COVID-19 case series and disease-based studies. However, in the absence of a US national healthcare system, most longstanding US epidemiology studies are not able to establish comprehensive linkages to electronic health records (EHRs) and have depended on medical records ascertainment and adjudication for cardiovascular and respiratory health outcomes. A protocol to define hospitalizations for COVID-19 among epidemiology cohorts was lacking.

The Collaborative Cohort of Cohorts for COVID-19 Research (C4R) study is a unique meta-cohort of NIH-funded prospective, observational epidemiology cohort studies that was funded to perform standardized, prospective ascertainment of COVID-19, including physician adjudication of medical records for COVID-19 hospitalizations and deaths (hereafter, “events”). The primary purpose of this report is to describe the C4R events adjudication protocol and preliminary experience applying the protocol from February 2021 to June 2023. This report includes a comparison of ICD-based versus physician adjudicated protocol-based classification for COVID-related fatal and non-fatal hospitalizations.

## Materials and methods

### Study design

C4R enrolled adult participants from 14 long-standing cardiovascular, neurological, and respiratory cohorts [4]: Atherosclerosis Risk in Communities (ARIC) [5, 6], Coronary Artery Risk Development in Young Adults (CARDIA) [7], Genetic Epidemiology of COPD (COPDGene) [8], Framingham Heart Study (FHS) [9], Hispanic Community Health Study/Study of Latinos (HCHS/SOL) [10–13], Jackson Heart Study (JHS) [14–16], Mediators of Atherosclerosis in South Asians Living in America (MASALA) [17, 18], Multi-Ethnic Study of Atherosclerosis (MESA) [19], Northern Manhattan Study (NOMAS) [20], Prevent Pulmonary Fibrosis (PrePF) [21], REeasons for Geographic and Racial Differences in Stroke (REGARDS) [22], Severe Asthma Research Program (SARP) [23], Subpopulations and Intermediate Outcome Measures in COPD Study (SPIROMICS) [24], and the Strong Heart Study (SHS) [25, 26]. Details on each of the component cohorts are provided in S1 Appendix and S1 Table. Cohort participants previously consented to in-person, telephone, and/or e-mail contact and for abstraction of medical records.

C4R received funding in 2020 to perform standardized prospective data collection on COVID-19 and to harmonize pre-pandemic deep phenotyping available in the cohorts. Columbia University developed the standard questionnaires and protocols and served as the Data Coordination and Harmonization Center (DCHC) for C4R (Columbia University Institutional Review Board, AAAT3035). Of note, in some cohorts, COVID-19 questionnaires were initiated before the development of a standard C4R questionnaire; these questionnaires were later harmonized with the C4R questionnaire and classified as C4R questionnaires.

As previously reported [4], following a cohort ancillary studies model, researchers in each cohort study were directly responsible for accomplishing data collection in accordance with the standard protocols and under the supervision of their own observational studies monitoring board, steering committee, institutional review board (IRB), and any other applicable regulatory authorities. Columbia University served as the Data Coordination and Harmonization Center (DCHC) for C4R (Columbia University Institutional Review Board, AAAT3035). A full list of cohort IRBs supervising implementation of the C4R protocols is provided in S2 Table.

Following cohort-specific IRB approval and consent processes (including verbal, remote, and traditional written informed consent), adult participants in the pre-existing cohort studies were enrolled into the C4R ancillary study on a rolling basis from April 9, 2020, to February 28, 2023. COVID-19 data collection was accomplished by two waves of questionnaires (Wave 1: April 2020–May 2022; Wave 2: February 2021–February 2023), SARS-CoV-2 serosurvey (February 2021–February 2023), and ascertainment and adjudication of COVID-19 events, which are the subject of this report.

For the purposes of the work presented in this manuscript, data were accessed at Columbia University from July 1, 2021, to October 1, 2023.

### Protocol development and coordination

The C4R events protocol is included in S2 Appendix. The protocol was developed by investigators and cohort personnel with experience in clinical events ascertainment [5, 7, 9, 11, 12, 17, 19, 20, 26–33] and approved by the C4R Cohort Coordinating Committee (CCC) in February 2021. Relevant administrative and review forms were coded into the C4R Events REDCap [34, 35] toolkit, which was made available to the cohorts via a central (Columbia) instance or for local adaptation by cohort data coordinating centers (DCCs). For the central instance, cohort-specific Data Access Groups (DAGs) ensured that each cohort was only able to enter or access its own participant data. Cohorts provided bimonthly tracking reports regarding cohort-specific elements of protocol completion to the DCHC, which integrated these reports and generated status reports to the Events Subcommittee, the CCC, and funding agencies. De-identified COVID-19 events data were made available for analysis on the C4R Analysis Commons and shared with cohort-specific DCCs for cohort use and transfer to other data sharing platforms.

### COVID-19 events ascertainment

Potential COVID-19 hospitalizations and deaths were ascertained by cohorts via several mechanisms. C4R questionnaires, which were administered in two waves across all 14 cohorts, included questions regarding hospitalization for COVID-19 (S3 Table). If necessary (e.g., in cases of participant dementia or death), questionnaires were administered to a participant’s proxy. Additional COVID-19-related hospitalizations and deaths were identified by regular non-C4R follow-up calls conducted in 9 cohorts (ARIC, CARDIA, FHS, HCHS/SOL, JHS, MESA, NOMAS, REGARDS, SHS) to collect information on all-cause hospitalization and vital status. These data were supplemented with information collected at in-person exams, which were conducted in all cohorts during the pandemic period, except for NOMAS and REGARDS. Various non-questionnaire ascertainment methods, such as active surveillance of local EHR systems and other sources (e.g., obituaries), were performed by ARIC, FHS, JHS, SARP, SHS, and selected clinical sites in CARDIA, COPDGene, and MESA. Most cohorts supplemented vital status data with National Death Index (NDI) searches, although these are subject to reporting lags and were not available for the pandemic period at the time of this report.

After confirming participant/proxy consent, cohort staff requested copies of medical records for potential COVID-19-related hospitalizations and deaths, including physician notes (admission, consultation, discharge), radiology and laboratory reports, electrocardiogram reports, and discharge diagnoses (including discharge diagnosis ICD codes). Where applicable, death certificates were obtained. Medical records submitted for central review at the DCHC were de-identified prior to secure file transfer.

### Adjudication

#### Eligibility for adjudication

Hospitalizations (fatal and non-fatal) and out-of-hospital deaths were eligible for protocolized medical record review if they were assigned, in any position, any of the following COVID-19 discharge diagnoses based on the International Classification of Diseases, Tenth Revision (ICD-10), Clinical Modification codes [36]: confirmed COVID-19 (U07.1), post-infectious state after COVID-19 (U09.9), Multisystem inflammatory syndrome associated with COVID-19 (M35.81), Personal History of COVID-19 (Z86.16), Pneumonia due to coronavirus disease 2019 (J12.82), Other viral pneumonia (J12.89), or, for events occurring prior to May 1 2020, other coronavirus (B97.29). In the absence of these discharge diagnoses, records were considered eligible for review if there was evidence of a positive COVID-19 test, physician suspicion of COVID-19, or next-of-kin interview indicating suspected or known COVID-19 infection. Episodes of treatment in the Emergency Department (ED) for >24 hours were classified as hospitalizations since many hospitals used the ED for inpatient care during the heights of COVID-19 surges.

#### Adjudication process

Eligible COVID-19-related hospitalizations (fatal and non-fatal) and out-of-hospital deaths were subjected to adjudication by physicians and/or clinical nurse practitioners with experience in evaluating hospitalized COVID-19. Reviewers were trained via webinar and individualized instruction. Three cohorts (FHS, REGARDS, SARP) elected to perform review by local adjudicators; records from the remaining cohorts were reviewed centrally at the DCHC. Adjudication entailed data abstraction, including information on oxygenation levels and medication administration, followed by classification of COVID-19 outcomes. All COVID-19 outcomes were defined as definite, probable, or absent, based on specific criteria, including symptoms and test results, and adjudicator judgment (S4 Table).

#### Adjudication of COVID-19 infection

Adjudication of Definite COVID-19 Infection required evidence of a positive SARS-CoV-2 test. Per protocol, an administrative criterion (i.e., assignment of ICD U07.1) was sufficient to classify Definite COVID-19 infection, since this diagnosis code specifically requires positive testing for SARS-CoV-2; of note, for all other outcomes, administrative criteria were insufficient for definite classification but could be used to justify a probable classification. Definite COVID-19 infection was necessary to define any other COVID-19 outcome as definite. Adjudication of Probable COVID-19 Infection required anticipated signs and symptoms of COVID-19 without confirmatory testing. In the absence of adjudicated Definite or Probable COVID-19 infection, no additional outcomes were adjudicated.

#### Adjudication of COVID-19 hospitalization

In the context of Definite or Probable SARS-CoV-2 infection, hospitalization “due to” COVID-19 was defined as being admitted to the hospital for COVID-19-related signs or symptoms; developing COVID-19-related signs or symptoms during hospitalization; or, death in the ED. Whereas, hospitalization “with” COVID-19 was defined as being diagnosed with SARS-CoV-2 infection, yet being admitted to the hospital for a reason other than COVID-19 related signs or symptoms, without developing COVID-19 signs or symptoms during hospitalization.

#### Adjudication of COVID-19 severity

Definitions of severe and critical disease were based on NIH COVID-19 treatment guidelines [37].

#### Adjudication of COVID-19 complications

While treating physician notes and administrative criteria could be used for Probable classification of COVID-19 complications, Definite classification of COVID-19 pneumonia, pulmonary embolus (PE), deep vein thrombosis (DVT), or stroke required radiologic evidence in the medical record. Definite classification of acute kidney injury (AKI) was based on reported blood creatinine levels or initiation of renal replacement therapy. Definite COVID-19 myocardial infarction (MI) was defined based on biomarker and electrocardiographic or pathologic criteria to identify cases of MI caused by acute atherothrombotic coronary artery disease, or “Type 1” MI, in the context of COVID-19 infection [38]. Probable COVID-19 MI was defined more broadly and likely captures Type 1 and Type 2 MI. Myocardial injury was defined as a maximum recorded troponin level that was greater than two times the upper limit of normal (ULN), with or without associated ischemic symptoms.

#### Re-adjudication

Following completion of adjudication, reviewers could request a second independent adjudication for challenging cases. A random 10% subset of records was also submitted for a second independent adjudication.

#### Analysis

Characteristics of C4R participants, with and without an ascertained or adjudicated COVID-19 event, were tabulated. The incidence of events ascertained and adjudicated per month (based on date of event) was plotted. Since we found that records relating to out-of-hospital deaths (e.g., death certificates) did not contain sufficient data for adjudication, the following analyses were limited to adjudicated fatal and non-fatal COVID-19 hospitalizations among participants who consented to data sharing on the C4R Analysis Commons. The incidence of COVID-19-related outcomes was tabulated, by level of certainty. Interrater agreement was assessed via positive agreement, negative agreement, and the Cohen’s κ-statistic [39]. The performance of discharge diagnosis ICD code-based classification was compared against definite C4R classifications, which were treated as the reference standards; probable classifications were excluded from these comparisons because they included discharge diagnosis ICD codes in the diagnostic criteria. Sensitivity, specificity, positive predictive value (PPV) and negative predictive value (NPV) were calculated. Also, myocardial injury was compared to adjudicated classifications of definite COVID-19 MI. Analyses were performed in SAS Studio software (SAS Institute, Cary, NC) on the C4R Analysis Commons.

## Results

### COVID-19 events ascertainment

**Fig 1:**
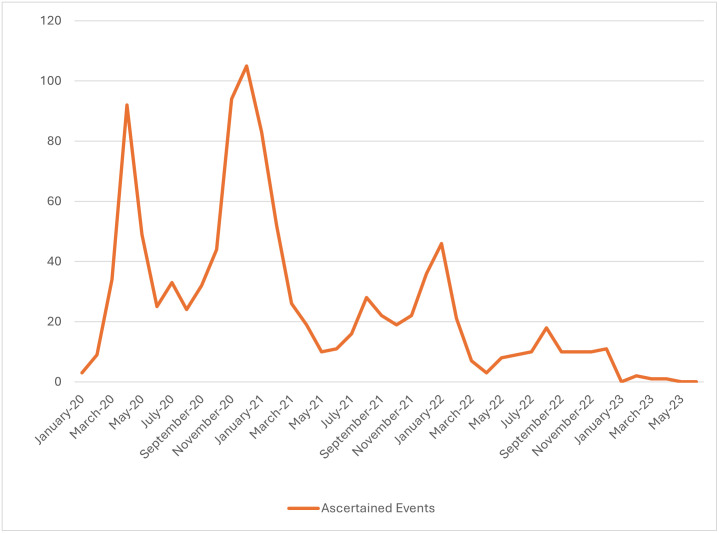
Incidence of COVID-19-related hospitalization and/or death over C4R follow-up, United States, January 2020—June 2023. Incidence is calculated per month and based on date of event.

Among 49,790 C4R participants, 1,974 potential COVID-19-related hospitalization and/or deaths among 1,772 (3.6%) participants were ascertained between January 2020 and June 2023 (Fig 1), for an estimated incidence density rate of 11.9 per 1,000 person-years of follow-up. Overall, the cohorts reported that half (53%) of these events were identified by a C4R questionnaire. As of June 2023, 1,768 (90%) medical records were requested, of which 1,523 (86%) were obtained. The most common reasons for failure to obtain medical records were inability to obtain participant consent and lack of response from the hospital.

**Fig 2:**
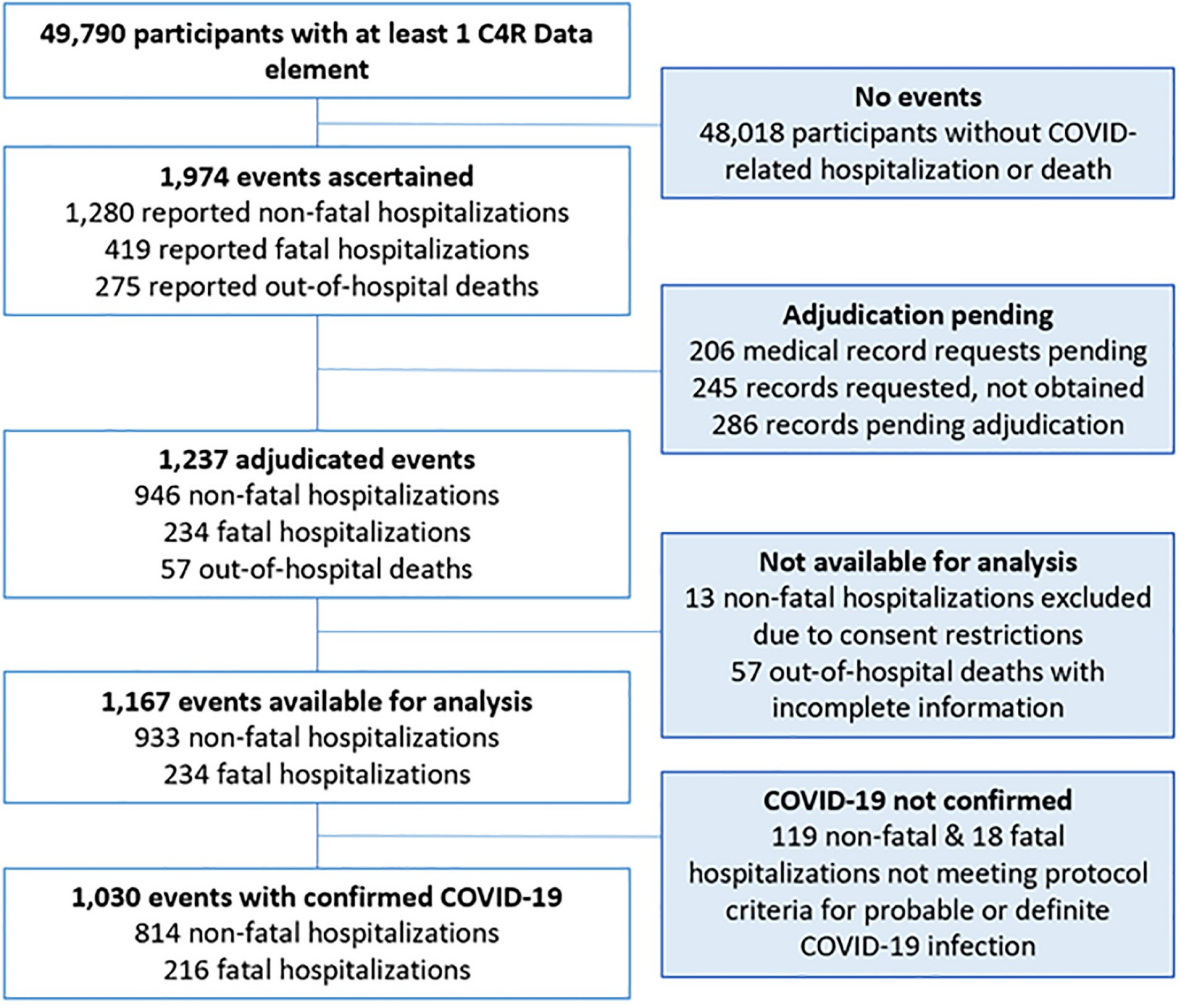
Consort diagram of participants with COVID-19-related hospitalizations and deaths ascertained by C4R, January 2020—June 2023.

Following 3 years of protocol funding (October 1, 2020—October 1, 2023), 1,237 of 1,974 (63%) events were adjudicated over 28 months. After exclusion of 57 out-of-hospital deaths due to incomplete information and 13 non-fatal hospitalizations with consent restrictions, there were 1,167 (59% of the 1,974) events available for analysis, of which 1,030 had evidence of COVID-19 (88% of 1,167 events available for analysis) (Fig 2).

Table 1 compares socio-demographic and clinical characteristics of 1,772 participants among whom a potential COVID-19 hospitalization was ascertained, including those for whom events were (N = 1098) and were not yet (N = 674) adjudicated as of October 2023, versus those with no COVID-19 hospitalization or death ascertained through June 2023 (N = 48,018). Compared to participants without a potential COVID-19 hospitalization or death, those with an ascertained event were more likely to be male, to report American Indian ancestry, and to have a history of pre-pandemic smoking, diabetes, or hypertension; they were less likely to have education beyond college and less likely to be vaccinated compared to those without ascertained events. Notably, among participants with ascertained potential COVID-19 events, characteristics of those with adjudicated records were generally similar to those pending adjudication. More than one COVID-19 hospitalization was ascertained in 50 (3%) participants.

**Table 1:** Baseline characteristics of C4R participants according to ascertainment and adjudication of COVID-19 hospitalization or death.

Characteristic	No event as of June 30, 2023		Ascertained event as of June 30, 2023; pending adjudication as of October 1, 2023		Adjudicated event	
	N	%^b^	N	% ^b^	N	% ^b^
Participants^a^, n (%)	48018		674		1098	
COVID-19 hospitalizations and deaths, n			737		1237	
Sex						
Male	19932	42	304	48	456	46
Female	28019	58	326	52	543	54
Race/Ethnicity						
Asian	1136	2	9	1	8	1
American Indian and Alaskan Native	1456	3	38	6	248	23
Black	10733	22	210	33	209	19
White	22347	47	174	27	457	43
Hispanic	12192	25	207	32	248	23
Other	76	0	1	0	0	0
Age						
18–30 years	565	1	5	1	3	0
31–64 years	16673	35	167	28	182	21
65+ years	29888	63	417	71	695	79
Cohort						
ARIC	5560	12	53	8	311	28
CARDIA	2798	6	29	4	27	2
COPDGene	4109	9	33	5	49	4
FHS	3300	7	15	2	52	5
HCHS/SOL	11021	23	132	20	191	17
JHS	2279	5	100	15	0	0
MASALA	572	1	1	0	0	0
MESA	3268	7	189	28	47	4
NOMAS	909	2	23	3	67	6
PrePF	622	1	3	0	3	0
REGARDS	10126	21	20	3	179	16
SARP	508	1	2	0	4	0
SPIROMICS	1553	3	23	3	9	1
SHS	1393	3	51	8	159	14
Geographic Region						
Middle Atlantic	7804	16	145	22	274	25
Midwest	9605	20	140	21	154	14
New England	3162	7	15	2	56	5
South	16155	34	210	32	297	28
Southwest	857	2	5	1	3	0
West	3500	7	63	10	242	22
Smoking Status						
Current	6610	14	97	16	128	13
Former	17258	37	247	40	413	43
Never	23339	49	270	44	413	43
Body mass index						
<25 kg/m^2^	11115	24	90	15	169	18
25–29.9 kg/m^2^	17031	37	210	35	309	33
30–34.9 kg/m^2^	10727	23	165	27	262	28
>35 kg/m^2^	7493	16	139	23	206	22
Education						
Less than high school	5608	12	109	18	178	19
High school	10820	23	151	25	273	29
College	9081	20	108	18	180	19
Beyond college	20969	45	239	39	316	33
Medical History						
Diabetes	9099	19	173	28	301	31
Hypertension	23580	50	380	62	605	63
Cardiovascular disease	4712	11	80	14	165	19
Chronic obstructive pulmonary disease	3361	9	76	13	82	12
Asthma	4856	14	73	13	119	17
Received COVID-19 vaccine	28837	91	265	87	455	82
Infection wave during which event occurred						
First wave (WT, NE)	1133	2	155	23	173	16
Second wave (WT, rest of US)	573	1	100	15	136	12
Third wave (Alpha, winter)	1487	3	159	24	338	31
Fourth wave (MI, spring)	385	1	42	6	45	4
Fifth wave (Delta)	809	2	64	10	131	12
Sixth wave (Omicron)	904	2	17	2	45	4
Unknown wave	1093	2	87	13	191	17
No infection confirmed	41634	87	50	7	39	4

ARIC = Atherosclerosis Risk in Communities Study; CARDIA = Coronary Artery Risk Development in Young Adults, COPDGene = Genetic Epidemiology of Chronic Obstructive Pulmonary Disease; FHS = Framingham Heart Study; HCHS/SOL = Hispanic Community Health Study/Study of Latinos; JHS = Jackson Heart Study; MASALA = Mediators of Atherosclerosis in South Asians Living in America; MESA = Multi-Ethnic Study of Atherosclerosis; NOMAS = Northern Manhattan Study; PrePF = Preclinical Pulmonary Fibrosis; REGARDS = Reasons for Geographic and Racial Differences in Stroke; SARP = Severe Asthma Research Program; SPIROMICS = Subpopulations and Intermediate Outcome Measures in COPD Study; SHS = Strong Heart Study.

^a^Multiple events per participant were possible which is why the number of events is greater than the number of participants. There were missing data for some pre-pandemic characteristics, in which case percentages are reported based on the number of non-missing observations.

^b^Column percentages reported.

### Adjudication

#### Adjudication process

There were 7 reviewers at the C4R DCHC, 5 at FHS, 3 at REGARDS, and 2 at SARP. The median length of medical records was 59 pages (IQR: 37, 105). The average time to review was approximately 30 minutes per record.

#### Adjudicated outcomes

Definite SARS-CoV-2 infection was adjudicated in 87% of 1,167 events, whereas probable infection was adjudicated in only 1%. Among 1,030 adjudicated hospitalized events in which SARS-CoV-2 infection was definite or probable, COVID-19 illness was diagnosed as the cause of 952 (93%) of the hospitalizations. Table 2 describes the adjudicated probable and definite diagnoses of SARS-CoV-2 infection, acute COVID-19 illness severity, and acute COVID-19 complications following the criteria described in S3 Table: 77% were adjudicated as severe COVID-19, 31% as critical COVID-19, 80% had adjudicated COVID-19-associated pneumonia, and 34% had adjudicated COVID-19-associated AKI. Adjudicated fatal hospitalization due to COVID-19 and other cardiopulmonary complications such as PE, DVT, and MI were less common.

**Table 2:** Adjudicated COVID-19 outcomes for COVID-19-related hospitalizations ascertained by C4R.

Outcomes	Eligible for Adjudication	Certainty Level of Adjudication^a^					
		Definite		Probable		Definite or probable	
	N	N	%	N	%	N	%
COVID-19 infection	1167	1013	87	17	1	1030	88
Hospitalization due to COVID-19	1030	913	89	39	4	952	92
Severe COVID-19 illness	1030	726	70	65	6	791	77
Critical COVID-19 illness	1030	275	27	44	4	319	31
Fatal COVID-19 hospitalization	1030	194	19	16	2	210	20
Pneumonia caused by COVID-19	1030	711	69	111	11	822	80
Renal failure caused by COVID-19	1030	240	23	110	11	350	34
PE caused by COVID-19	1030	32	3	16	2	48	5
DVT caused by COVID-19	1030	31	3	2	0	33	3
Stroke caused by COVID-19	1030	13	1	12	1	25	2
Myocardial infarction caused by COVID-19^b^	1030	7	1	43	4	50	5

^a^Percentages displayed are of the total number of events eligible for adjudication of the COVID-19-related diagnosis.

^b^Definite COVID-19 myocardial infarction (MI) was defined based on biomarker and electrocardiographic or pathologic criteria to identify cases of MI caused by acute atherothrombotic coronary artery disease, or “Type 1” MI, in the context of COVID-19 infection. Probable COVID-19 MI was defined more broadly and likely captures Type 1 and Type 2 MI.

#### Adjudicator agreement

Of 139/1237 (11%) hospitalizations that underwent a second adjudication, there was almost perfect agreement for adjudication of COVID-19 infection, critical illness, stroke, PE, DVT, and fatal hospitalization (Table 3). There was also strong agreement for hospitalization, severe illness, pneumonia, and renal failure, and moderate agreement for MI.

**Table 3:** Interrater agreement for adjudication of COVID-19 outcomes in C4R.

Outcome	Agreement		Disagreement^c^	K-statistic	95% CI
	Positive ^a^	Negative ^b^			
COVID-19 infection	129	9	1	0.94	0.83–1.00
Hospitalization due to COVID-19	120	15	3	0.90	0.78–1.00
Severe COVID-19 illness	89	40	9	0.85	0.76–0.94
Critical COVID-19 illness	40	94	3	0.95	0.89–1.00
Fatal COVID-19 hospitalization	25	110	2	0.95	0.89–1.00
Pneumonia caused by COVID-19	102	29	7	0.86	0.76–0.96
Renal failure caused by COVID-19	45	88	6	0.90	0.83–0.98
PE caused by COVID-19	8	130	0	1.00	1.00–1.00
DVT caused by COVID-19	4	134	0	1.00	1.00–1.00
Stroke caused by COVID-19	4	134	0	1.00	1.00–1.00
Myocardial infarction caused by COVID-19	4	128	5	0.60	0.28–0.92

CI = confidence interval.

^a^Positive agreement was defined as both reviewers confirming the outcome with either probable or definite certainty.

^b^Negative agreement was defined as both reviewers classifying the outcome as neither probable nor definite.

^c^Disagreement was defined as one reviewer classifying the outcome as probable or definite, and the other reviewer classifying the outcome as neither probable nor definite.

#### Comparison of adjudication vs. ICD codes

Compared to adjudicated diagnoses with a definite certainty level, discharge ICD code-based classification was sensitive and specific for pneumonia (sensitivity = 84%, specificity = 90%), but less sensitive (57–81%) for cardiovascular and renal complications (Table 4). The PPV of ICD-based classification for COVID-19-related pneumonia (J12.82, pneumonia due to coronavirus disease 2019, J12.89, other viral pneumonia), AKI (N17, Acute kidney injury), PE (ICD Code I26, PE), DVT (I82, DVT) and stroke (I63, Cerebral infarction) was excellent, ranging from 81–97%; however, the PPV for COVID-19-related MI (I21, Acute myocardial infarction) was 33%.

**Table 4:** Sensitivity, specificity, and predictive values for discharge diagnosis codes versus adjudicated definite COVID-19 outcomes.

Discharge diagnosis ICD code, Label	Reference standard (adjudicated outcome)	Agreement and disagreement of discharge diagnosis code with adjudicated definite outcome, N				Performance of discharge diagnosis code versus adjudicated definite outcome, %			
		ICD code confirmed by review	False positive by ICD code vs review	False negative by ICD code vs review	Outcome not confirmed by ICD or review	Sensitivity	Specificity	PPV	NPV
J12.82, Pneumonia due to coronavirus disease 2019 J12.89, Other viral pneumonia	Pneumonia caused by COVID-19	578	19	110	170	84%	90%	97%	61%
N17, Acute kidney injury	Renal failure caused by COVID-19	146	23	88	617	62%	96%	86%	88%
I26, Pulmonary embolism	PE caused by COVID-19	26	3	6	926	81%	100%	90%	99%
I82, Deep venous thrombosis	DVT caused by COVID-19	21	5	9	937	70%	99%	81%	99%
I63, Cerebral infarction	Stroke caused by COVID-19	8	0	5	952	62%	100%	100%	99%
I21, Acute myocardial infarction	Myocardial infarction caused by COVID-19	4	8	3	920	57%	99%	33%	100%

^a^Table does not include comparisons between ICD-based classification and adjudicated diagnoses with a certainty level of ‘probable.’ Records without an assigned discharge diagnosis code are also excluded.

DVT = deep venous thrombosis; ICD = international classification of diseases; MI = myocardial infarction; NPV = negative predictive value; PE = pulmonary embolism; PPV = positive predictive value.

#### Comparison of adjudicated MI vs. myocardial injury

A substantial number of participants with adjudicated events had myocardial injury that was not adjudicated as COVID-19-related MI. Among the 1,030 hospitalized events in which SARS-CoV-2 infection was adjudicated as definite or probable, 170 (25%) demonstrated myocardial injury. Of these 170 events with myocardial injury, 25 (15%) were assigned an ICD code for MI, 5 (3%) were adjudicated as definite COVID-19-related MI, and 38 (22%) were adjudicated as probable COVID-19-related MI; the majority (75%) were not adjudicated as definite or probable COVID-19-related MI. Of note, these groups are not directly comparable with the groups described in Table 4, which only included events with ICD codes available and excluded events with probable MI diagnoses.

## Discussion

Protocolized adjudication confirmed four out of five hospitalizations for COVID-19 in a US meta-cohort of prospective epidemiology cohorts and adjudicated cases of pneumonia, PE, and other conditions that were not indicated by discharge diagnosis codes. These results illustrate the importance of systematic medical record review for robust classification of COVID-19 outcomes in epidemiology cohort studies, which provide unique opportunities to study antecedent risk factors for acute COVID-19 and post-COVID conditions.

Over three years, C4R ascertained at least one potential COVID-19-related hospitalization or out-of-hospital death in 3.6% of participants, equivalent to an incidence per 1,000 person-years of follow-up of 11.9. For comparison, in 8 of the C4R cohorts, the incidence per 1,000 person-years for atherosclerotic cardiovascular disease events has been estimated at 9.7 [40]. Hence, the results of C4R events ascertainment to date highlight the major impact of COVID-19 on cohort participants and provide a substantial, longitudinal dataset to support well-powered analyses of risk factors and sequelae.

Nonetheless, our experience highlights certain limitations of standard cohort events surveillance for ascertainment of COVID-19-related events. Of hospitalizations that were ascertained as potentially COVID-19-related, SARS-CoV-2 infection could not be confirmed in one in eight events. These cases could have been due to incorrect self-reporting of a COVID-19 hospitalization on a C4R or cohort questionnaire, or active surveillance methods that were more sensitive than specific. Of note, some cases where infection could not be confirmed may have been true infections, but the medical records lacked sufficient detail for adjudication. Hence, rather than censoring all non-confirmed events, C4R is assigning a certainty level for all its COVID-19 outcomes, so that investigators can select the outcome most suitable for their specific research needs.

Our findings also demonstrate the limitations of discharge diagnosis codes to define hospitalization “for” versus “with” SARS-CoV-2 infection. Of hospitalizations with confirmed SARS-CoV-2 infection, symptoms of COVID-19 illness did not contribute to the hospitalization in 7% of cases. These findings are similar to prior reports examining the role of SARS-CoV-2 in hospitalizations using EHR systems. Adjudicated C4R outcomes will allow investigators to exclude these cases of hospitalization with incidental COVID-19 from studies designed to examine risks and sequelae of severe COVID-19 illness.

Furthermore, we found that discharge diagnosis codes were insensitive for cardiopulmonary and renal complications. We adjudicated a substantial number of cases of COVID-19 pneumonia, AKI, and MI among records without a corresponding discharge diagnosis code. This may be related, in part, to the major challenges of charting during COVID-19 surges, when documentation standards were modified to prioritize direct patient care activities. Nonetheless, before the pandemic period, previous investigations on the use of administrative data to identify cases of pneumonia have found that ICD codes are imprecise and can result in a substantial number of pneumonia cases going undetected [41, 42]. These results are supported by earlier findings that ICD-10 code N17 often misses AKI during hospitalizations for kidney transplant patients [43]. Potential misestimation of the incidence of MI using claims or administrative data has also been well-described [44–47]. This has been one justification for longstanding—albeit labor- and time-intensive—ASCVD events adjudication programs in many of the C4R cohorts.

As expected, we found that a substantial proportion of hospitalized events included evidence of myocardial injury, defined by troponin values two times greater than the upper limit of normal; however, only a small subset of these cases was adjudicated as COVID-19 MI. We elected to restrict definite MI to confirmed cases of type 1 (ST-elevation MI) or type 3 MI (MI resulting in death with pathological evidence of MI). It may be difficult to differentiate type 2 MI/supply-demand mismatch, which requires documentation of a rise and fall in troponin levels and symptoms of ischemia, from myocardial injury (at least one elevated troponin level), based solely on review of obtained medical records. Prior studies demonstrated that elevated troponin values are common in patients hospitalized with COVID-19 [48–50], and may occur with conditions other than MI, such as cardiomyopathy, acute cor pulmonale, arrhythmias, or cardiogenic shock [51]. Our results suggest that the true incidence of type 1 MI, due to plaque disruption and thrombosis leading to coronary occlusion, was rare in the context of acute COVID-19, supporting the utility of protocolized adjudication in validating these outcomes for COVID-19 cardiovascular research.

Altogether, our findings have several implications for EHR-based studies that do not adjudicate endpoints. Our results suggest that ICD-based events definitions could overestimate the number of cases of hospitalization due to COVID-19 illness and underestimate COVID-related complications. In addition to incorrectly estimating the prevalence and incidence of COVID-related outcomes, the observed measurement errors could reduce the robustness and reproducibility of epidemiologic analyses. If ICD misclassification was nondifferential according to risk factors of interest, it would reduce precision and increase the risk of type 2 error; if misclassification was associated with a risk factor of interest, this could potentially bias results away from the null hypothesis. Of note, EHR-based studies often use complex algorithms to define clinical outcomes and do not rely on ICD codes alone. Nonetheless, our findings underscore the importance of validating outcome definitions via comparison with robust approaches such as protocolized events adjudication.

### Strengths and limitations

Strengths of this study include prospective ascertainment of potential COVID-19-related hospitalizations and deaths in a well-characterized, multi-ethnic, US community-based sample of adults that is relatively free of referral, survival, and recall biases compared with studies that only include hospitalized patients. The adjudication protocol, which was modeled on gold-standard epidemiology cohort events adjudication in many of the C4R cohorts, was fully standardized across the cohorts and implemented at scale to generate robust events data for analysis within three years of program initiation.

Nonetheless, certain limitations must be considered, in addition to those noted above. Medical records have not yet been obtained for 37% of cases due to lack of participant consent, lack of response from hospital systems, or other operational delays that are common to cohort events ascertainment operations. This highlights the need for novel approaches for cohorts to access medical records, such as the consenting of participants to share their own electronic medical records—now available to patients via the 21^st^ Century Cures Act [52]—with cohorts [52]. The characteristics of participants with missing versus available medical records in C4R were comparable, so there was no clear evidence of selection bias. Some medical records had incomplete data available for adjudication, which could be related to relaxation of documentation requirements during the pandemic. Few out-of-hospital deaths were ascertained, and medical records for out-of-hospital deaths were often incomplete, due to various reasons, including lack of court documents from the family of the deceased to obtain records, or hospital decision to not grant access to the decedent’s medical records for research purposes. To address this limitation, additional information on out-of-hospital deaths associated with COVID-19 will be obtained from the NDI, which provides complete ascertainment of deaths in the US, including ICD-codes; unfortunately, there are substantial time lags inherent in NDI reporting.

## Conclusions

Ascertainment and adjudication of COVID-19 hospitalizations and deaths in longstanding NIH-funded cohort studies were feasible, albeit time- and resource-intensive, and our results illustrate the importance of systematic medical records adjudication for robust classification of COVID-19 events. Adjudication confirmed SARS-CoV-2 infection in 88% of ascertained events and found that infection may have been incidental to the hospitalization in 7% of the cases. Compared to adjudication, discharge diagnosis codes were insensitive for acute cardiovascular and renal complications of COVID-19. Novel approaches to expedite medical records access and linkage would augment unique opportunities for COVID-19 and other health outcomes research, particularly for emerging diseases, in NIH-funded epidemiology cohorts.

## Supporting information

S1 Appendix Cohort descriptions. (DOCX)

S2 Appendix C4R events protocol. (PDF)

S1 Table Characteristics of participants in C4R cohorts, United States, March 1, 2020. (DOCX)

S2 Table Cohort Institutional Review Boards (IRBs) supervising implementation of the C4R protocols. (DOCX)

S3 Table Selected COVID-19 questionnaire elements and their inclusion by cohorts in questionnaires for C4R, United States, April 2020–February 2023. (DOCX)

S4 Table Criteria for classification of COVID-19 diagnoses as definite or probable based on medical record review. (DOCX)
