# Supplementary material for: Classifying COVID-19 hospitalizations in epidemiology cohort studies: The C4R study
Source: PLoS One. 2025 Feb 10;20(2):e0316198. doi: 10.1371/journal.pone.0316198 (PMC11809881; doi:10.1371/journal.pone.0316198)
Supplement: S2 Appendix — (PDF) [file pone.0316198.s002.pdf]

[redacted] Collaborative
Cohort of Cohorts
for COVID-19 Research

Events Ascertainment Protocol

Contents

Section 1: Introduction.....2
Section 2: Cohort and C4R roles and responsibilities.....3
Section 3: Reporting COVID-19 test results occurring outside of a COVID hospitalization.....4
Section 4: Identification of potential COVID-related events.....5
Section 5: Obtaining medical records for hospitalized events.....7
Section 6: Obtaining information on out-of-hospital deaths.....8
Section 7: Defining eligibility for abstraction.....9
Section 8: Event Review Process.....10
COVID Test Result Form.....13
Initial Notification of COVID-related Event (Notification) Form.....14
ICD Form.....15
Review Form.....16
PASC Review Form.....23
Diagnosis Form: If Event Type = Hospitalization, PASC event, or deceased in emergency department...28
Diagnosis Form: If Event Type = Out of Hospital Death.....32

Section 1: Introduction

The following manual describes the protocol for identifying, documenting, reporting, and classifying COVID-related events for C4R.

The events of primary interest for C4R events review are:

- COVID-19 infection
- COVID-19 test positivity
- COVID-19 hospitalization
- COVID-19 severe illness
- COVID-19 critical illness
- COVID-19 complications
  - Pneumonia
  - Myocardial injury
  - Cerebral infarction
  - Pulmonary embolism
  - Deep venous thrombosis
  - Kidney injury
- Fatal COVID-19

This protocol focuses on the ascertainment of hospital and death records for identification of COVID-19 events. The protocol can be adapted for other types of medical records (e.g., nursing home, outpatient); however, since ascertainment of other records is not part of the funded scope of work for C4R, only hospital and death records will be discussed in this protocol.

Section 2: Cohort and C4R roles and responsibilities

Cohort staff will be responsible for the following activities:

- Identifying potential COVID-related events
- Obtaining medical records or death certificates for potential events
- Recording ICD codes from medical records or death certificates received for the events
- Determining eligibility of events for review
- For cohorts that elect to have records classified centrally by C4R, cohort staff will also be responsible for de-identifying medical records and death certificates according to cohort standards and transmitting them via secure file transfer to the C4R data coordination and harmonization center (DCHC).

Review will be performed by personnel with clinical experience (MD, RN, or equivalent) at the cohort level; alternatively, classification of de-identified records can also be performed by the C4R data coordination and harmonization center (DCHC).

Training materials for involved research personnel will be developed. A teleconference will be organized to review procedures and forms.

The DCHC will provide central coordination and operational support for all of the above activities.

Timelines

Cohort-specific data coordinating centers will be responsible for tracking ascertainment of potential COVID-related events. On a bi-weekly basis, the cohort data coordinating centers will be expected to report meta-data to the DCHC regarding the number of potential events that were identified.

"Closed" events datasets, defined as datasets of fully classified records, will be due on a quarterly basis (March 1, June 1, September 1, December 1). The target turnaround time from event identification to completion of the ICD form is < 2 months for events with electronic medical records available, and < 6 months for events subject to third party requests for medical records. For cohorts that request central DCHC abstraction of de-identified medical records, the target turnaround time from event identification to transfer to the DCHC will be set at the same intervals.

Modifications

Procedures will be piloted by the cohorts to identify any specific or general issues. Revisions to the protocol will be reviewed by the C4R Cohort Coordinating Committee.

Section 3: Reporting COVID-19 test results occurring outside of a COVID hospitalization

C4R encourages cohorts to request copies of positive COVID-19 tests reported by participants via questionnaire or other sources. A positive test result obtained outside of a hospitalization event will not be considered an "event" requiring notification, ICD, review, or diagnosis forms. Nonetheless, to capture data from "standalone" COVID-19 test results, which will be valuable to validate self-reported test results, cohort personnel should complete the "COVID test result" form upon receipt and review of the lab test report record from the participant.

Section 4: Identification of potential COVID-related events

Cohorts will conduct surveillance for potential COVID-related events via several complementary mechanisms, including:

- COVID-19 questionnaires: participants will complete at least two questionnaires relating to COVID-19 as part of C4R. Each of these questionnaires will include at least one question regarding whether the participant was hospitalized due to COVID-19 or related complications.
- Cohort events follow-up: some cohorts regularly contact participants regarding whether they have been hospitalized as part their events surveillance. To support C4R, cohorts are encouraged to assess for COVID-related hospitalization, if they are not already doing so.
- Participant/proxy notification: some cohorts request that participants notify the study if they are hospitalized. To support C4R, we recommend that cohorts encourage participants (via newsletter or other communication) to notify the study if they are hospitalized with COVID-19 or diagnosed with a non-hospitalized COVID-19 infection.
- Field center visits: some cohorts have field center examinations visits in the upcoming year(s). To support C4R, cohorts are encouraged to assess for COVID-related hospitalization at these visits, if they are not already planning to do so.
- Investigation of another type of potential event: some cohorts have established processes to investigate cardiovascular, pulmonary, and/or neurological events. When investigating one of these events, they may discover a COVID-19 infection.
- Obituary or other public notices: some cohorts may identify potential events via obituaries or other public notices.
- Electronic medical records (EMRs): some cohorts and sites have access to the EMRs of their participants. To support C4R, we encourage cohorts to assess health records that indicate probable or definite COVID-19 illness, as allowed by your IRB. Review of EMRs for ICD codes (Section 6) and/or manual review of records are encouraged. Options to use or expand current cohort EMR linkages are being developed.
- Local vital statistics searches or National death index (NDI) search: some cohorts periodically perform NDI searches for participants. This may identify deaths for which COVID-19 was recorded as the underlying or a contributing cause, although this will be limited by the delay in reporting.

Any information that cohort staff obtain regarding a potential COVID-related event should lead to the completion of an initial notification of COVID event form, by cohort staff or cohort specific coordinating center, regardless of source.

Every reported COVID hospitalization or death should thus have a notification form. Notification of a positive COVID-19 test result without a hospitalization or death does not require a notification form. If a participant has multiple hospitalizations, each one should have an INC form. A hospitalization leading to discharge and then out-of-hospital death should generate two INC forms, for (1) non-fatal hospitalization and (2) out of hospital death.

The INC event form includes the following elements:

- Participant ID
- Date of event: Month / Year
- Type of event
- How did the center find out about the event? -
- Status of medical/death records
- Notes (as needed)
- Reporter and date of report

This form will be included in a C4R REDCap toolkit for use or adaptation in C4R cohorts. Alternative forms for data capture can be used by cohorts, but standardized variable names and coding are strongly encouraged. The completed form should be transmitted to the cohort data coordinating center as soon as possible, and always within one week, following identification of the potential event. The cohort data coordinating center should submit a tracking report for cumulative INC forms on a bi-weekly basis to C4R.

If more than one INC form is submitted for the same participant, the records will be linked so that the relevant records can be reviewed together.

Section 5: Obtaining medical records for hospitalized events

Cohort staff will be expected to request the following medical records for all hospitalizations identified as potential COVID-related events:

- Discharge diagnoses and ICD codes (needed to determine eligibility, as defined below).
- ICD Code Sheet
- Notes
  - Admission note, History and Physical (H&P), HPI
  - ED note
  - Physician consult notes (all services)
  - ICU admission note (if applicable)
  - Discharge note/summary
- Death certificate (if applicable)
- Radiology reports:
  - Chest radiography (X-ray)
  - Chest computed tomography (CT)
  - Echocardiogram (TTE, TEE)
  - Head computed tomography (CT)
  - Brain magnetic resonance imaging (MRI)
  - Lower extremity ultrasound (Doppler/duplex)
- Laboratory reports:
  - Microbiology including COVID testing (PCR, antigen testing, and serology)
  - All other laboratory reports (of note, if it is possible to select only certain results: events review requires *only* creatinine, troponin, and arterial blood gas (ABG))
    - Note that the adjudication form includes the timing of which blood tests are needed. If records are being sent to the DCHC for adjudication, either send all the lab results (if that is easiest) or just sent the labs obtained at the time points in the adjudication form (e.g. lowest and highest creatinine, highest troponin, and initial ABG and ABG with the lowest paO_2)
- Medications
  - Please include home medication list (e.g., ACE inhibitors, anticoagulants)
  - Please include hospitalization medications listed in the adjudication form
- Vital signs (of note, if it is possible to select only certain vital signs, event review requires *only* respiratory rate, O2 saturation, O2 supplementation)
- Electrocardiogram report if suspected MI or new atrial fibrillation

<u>Examples of records that we do not need:</u>

- Progress notes, physical therapy notes, care planning notes, nursing notes

Cohorts with established events ascertainment protocols for other types of events (e.g., myocardial infarctions), should use the same procedures to obtain, store, and de-identify records. Under certain circumstances (e.g., when central review is requested), de-identified records will be transferred to the DCHC via secure file transfer protocols.

Section 6: Obtaining information on out-of-hospital deaths

For investigation of all deaths, and particularly out-of-hospital deaths, cohorts are strongly encouraged to obtain death certificates. ICD codes for COVID-19 in any position on a death certificate, with no additional information, can be used to classify COVID infection and fatal COVID-19.

Beyond the review of death certificates, cohorts may pursue additional means of investigation. Cohorts are encouraged to use their existing protocols for investigations of out-of-hospital deaths. These investigations often include a next-of-kin or other informant interview.

Section 7: Defining eligibility for abstraction

After submitting an INC form and obtaining medical records, death certificate, and/or a next-of-kin or other informant interview, cohort staff will determine whether a potential event is eligible for full COVID events classification.

To support this process, cohort staff will be expected to enter available ICD codes into an [ICD form].

Events meeting any of the following criteria should be classified as eligible for classification:

- ICD codes indicating COVID illness are sufficient to define eligibility for full abstraction.
  - U07.1, Confirmed COVID-19
  - U09.9, Post-infectious state after COVID-19
  - M35.81, Multisystem inflammatory syndrome associated with COVID-19
  - Z86.16, Personal History of COVID-19
  - J12.82, Pneumonia due to coronavirus disease 2019
  - J12.89, Other viral pneumonia
  - For events occurring prior to May 1, 2020:
    - B97.29, Other coronavirus (prior to May 1, 2020)
- In the absence of ICD codes (i.e., only if ICD codes are not available), the following criteria may be used to define eligibility:
  - Any diagnosis equivalent to one of the abovementioned ICD codes
  - Positive COVID test by PCR or antigen test, according to any of the following:
    - Physician note
    - Laboratory report
  - Negative COVID test in one of the following contexts:
    - Acute COVID-like illness (fever, cough, and/or shortness of breath without other identifiable cause) with suspected false-negative COVID testing (anticipated to be uncommon): this condition will be met if the discharge note documents suspected COVID despite negative test. When in doubt, such records should be considered eligible for review.
    - Post-acute sequelae of COVID-19: this condition will be met if the admission or discharge note documents prior COVID diagnosis, plus the conditions treated during the hospitalization are described as caused by, associated with, or linked to the prior COVID illness. When in doubt, such records should be considered eligible for review.
  - No COVID test available in one of the following contexts:
    - Acute COVID-like illness fever, cough, and/or shortness of breath without other identifiable cause) without confirmatory testing: this condition will be met if the discharge note indicates suspected COVID without confirmatory test. For events occurring between January 1 and March 1, 2020, symptoms of fever, cough, or shortness of breath without identifiable cause will be considered as potential events even if there is no physician documentation of suspected COVID.
    - Post-acute sequelae of COVID-19: this condition will be met if the admission or discharge note documents prior COVID diagnosis, plus the conditions treated during the hospitalization are described as caused by, associated with, or linked to the prior COVID illness. When in doubt, such records should be considered eligible for abstraction.
  - Next-of-kin or other informant interview indicating suspected or known COVID-19 infection

Of note, some patients receive multiple COVID-19 tests, and patients who have one or more negative COVID-19 tests followed by a positive test are considered to have a positive COVID-19 test.

Section 8: Event Review Process

Eligible hospitalized events will undergo classification using structured review forms. The reviewer will complete either (1) an acute COVID-19 form (for events with infection confirmed within 30 days of SARS-CoV-2 infection) or (2) a post-acute sequelae of COVID-19 (PASC) form (for events with infection confirmed 30 or more days prior to hospitalization). If there is ambiguity regarding whether to complete the acute or the post-acute review form, the reviewer should request guidance from the C4R Events Subcommittee. Both the review form and PASC form will be programmed in the C4R REDCap toolkit. It will include:

- Participant ID
- Event type
- COVID-19 test results
- Oxygen supplementation and advanced therapies
- Complications and relevant diagnostic testing

The structured forms will include the necessary and sufficient criteria for events classification, as summarized in the table below. Due to the nature of the details required for classification, the form will require clinical training to complete (MD, RN, or equivalent).

After completion of the acute or post-acute review form, the reviewer will be immediately prompted to complete a corresponding diagnosis form. This form will automatically indicate the diagnoses supported by the ICD form and the acute or post-acute review form. After reviewing the supported diagnoses, the reviewer will be asked to provide their final diagnoses.

If the reviewer's diagnosis is not in agreement with the supported diagnosis (generated algorithmically based on data entry on the ICD form and the acute or post-acute review form, this will trigger a second independent review. The first reviewer may also recommend a second review. Furthermore, 10% of records will be randomly sampled for a second review for quality control purposes. Reviewers will be retrained as needed.

Algorithm for classification of endpoints based on information provided in the ICD form and the review form:

Event	Probable	Definite
COVID-19 infection	Physician suspicion or informant suspicion of COVID-19<br><br>OR<br><br>Acute COVID-like illness (fever, cough, and/or shortness of breath without other identifiable cause) without positive COVID-19 test (includes no COVID-19 test and COVID-19 test believed to be false-negative)	Any of the following ICD:<br>U07.1, Confirmed COVID-19<br>U09.9, Post-infectious state after COVID-19<br>M35.81, Multisystem inflammatory syndrome associated with COVID-19<br>J12.82, Pneumonia due to coronavirus disease 2019<br><br>OR<br><br>Positive PCR or antigen test documented in medical record

COVID-19 hospitalization	Probable infection<br><br>AND<br><br>Admitted to hospital OR deceased in emergency department	Definite infection<br><br>AND<br><br>Admitted to hospital OR deceased in emergency department
COVID-19 severe illness	Probable infection<br><br>AND<br><br>Oxygen saturation < 94% on room air OR respiratory rate > 30 breaths/minute	Definite infection<br><br>AND<br><br>Oxygen saturation < 94% on room air OR respiratory rate > 30 breaths/minute OR requirement of BiPAP, high flow nasal cannula, mechanical ventilation, or ECMO
COVID-19 critical illness	Probable infection<br><br>AND<br><br>High-flow O2 OR intubation OR ECMO OR inotropes/pressors OR ICU or step down unit admission	Definite infection<br><br>AND<br><br>High-flow O2 OR intubation OR ECMO OR inotropes/pressors
COVID complications	Probable infection AND:	Definite infection AND:
Pneumonia	J12.82, Pneumonia due to coronavirus disease 2019<br>J12.89, Other viral pneumonia<br><br>OR<br><br>Physician documentation of pneumonia	Chest CT or CTA with pneumonia<br><br>OR<br><br>Chest x-ray with pneumonia
Myocardial infarction	ICD-10 I21<br><br>OR<br><br>Physician documentation of NSTEMI<br><br>OR<br><br>Physician documentation of STEMI	Maximum troponin > ULN<br><br>New ischemic electrocardiographic changes<br><br>Chest pain
Stroke	ICD-10 I63<br><br>OR<br><br>Physician documentation of stroke	CT head consistent with stroke<br><br>OR<br><br>MRI brain consistent with stroke
Pulmonary embolism	ICD-10 I26<br><br>OR	CTA indicative of PE<br><br>OR

	Physician note documenting presumptive PE with initiation of anticoagulation	V/Q indicative of PE
DVT	ICD-10 I82<br>Physician note documenting new venous thrombosis	Lower extremity Doppler showing DVT
Renal failure	ICD-10 N17<br><br>OR<br><br>Physician documentation of acute kidney injury (AKI)	Max/min or Max/baseline creatinine > 1.5<br><br>OR<br><br>New initiation of renal replacement therapy
Fatal COVID-19	Probable COVID-19 hospitalization ending in death<br><br>OR<br><br>Physician or other informant suspicion of COVID-19 contributing to death<br><br>OR<br><br>Acute COVID-like illness (fever, cough, and/or shortness of breath without other identifiable cause) without positive COVID-19 test within 28 days of death<br><br>OR<br><br>Judged by reviewer that COVID-19 is probable cause of death	Death certificate with ICD<br>U07.1, Confirmed COVID-19<br>U09.9, Post-infectious state after COVID-19<br>M35.81, Multisystem inflammatory syndrome associated with COVID-19<br>J12.82, Pneumonia due to coronavirus disease 2019<br><br>OR<br><br>Definite COVID-19 hospitalization ending in death<br><br>OR<br><br>Judged by reviewer that COVID-19 is definite cause of death

COVID Test Result Form

[redacted]

To be submitted for standalone COVID test results not occurring in association with a COVID hospitalization or death.

Participant ID: ______________________________

Test Date (Month / Day / Year): ___________________

What type of test was it? Pick one:

- Specimen type **Choose an item.**
- Diagnostic test type **Choose an item.**
    - If Other: ________

Test Result

☐ Positive or "Detected"
- If Positive:
    - Was "cycle count" or "viral load" reported? ☐ Yes ☐ No
    - If yes:
        - Cycle count or viral load (if available): ______________________
        - Units: ________________

☐ Negative or "Not Detected"

☐ Invalid or indeterminate

Notes (as needed):

Reporter: _______________________________

Date of report (month / day / year): ____________________

[redacted]

<u>Initial Notification of COVID-related Event (Notification) Form</u>

Please complete this form for each hospitalization or death event. If a participant has several consecutive hospitalizations, please complete a separate form for each hospitalization.

Note: This form may be redundant with a cohort specific form. Rather than fill out two forms, a cohort may decide that the cohort coordinating center will send the information to the DCHC every 2 weeks, rather than having the cohort field center staff will out this form. This needs to be standardized within each cohort.

- Participant ID: ____________________________
- Date of event (month / year): ___________________________
- Type of event (select one): **Choose an item.**
- How did the center find out about the event? Select all that apply:
  - ☐ C4R Questionnaire
  - ☐ Other cohort follow-up (including events surveillance)
  - ☐ Participant or proxy contacted the field/clinical center
  - ☐ Cohort field/clinical center visit
  - ☐ During investigation of another event
  - ☐ Obituary/local news
  - ☐ Electronic medical record surveillance or health information exchange
  - ☐ NDI or local vital statistics search
  - ☐ Other: ________________
- Status of medical/death records (select one): **Choose an item.**
  If you have access to the electronic medical record (i.e., you do not need to request records from the hospital), please mark "Obtained."
- Notes (as needed):
- Reporter: _____________________________
- Date of report (month / day / year): **Click or tap to enter a date.**

ICD Form

[redacted]

Please complete this form for each hospitalization or death event. If a participant has several consecutive hospitalizations, please complete a separate form for each hospitalization.

- Participant ID: ______________________________

Source of ICD codes (select one)

- ☐ ICD codes not available
- Discharge diagnoses (do not use admission diagnoses): **Choose an item.**
  - If selected, date of discharge: **Click or tap to enter a date.**
- Causes of death: **Choose an item.**
  - If selected, date of death: **Click or tap to enter a date.**

Please select any of the following COVID-event related ICD-10 codes (or, if ICD-10 codes are not provided, relevant diagnoses) listed on the record.
Codes/diagnoses that are eligibility criteria for C4R event review are underlined.

☐ U07.1, Confirmed COVID-19
☐ U09.9, Post-infectious state after COVID-19
☐ I21, Acute myocardial infarction
☐ I63, Cerebral infarction
☐ I26, Pulmonary embolism
☐ I82, deep venous thrombosis
☐ J12.82, Pneumonia due to coronavirus disease 2019
☐ J12.89, Other viral pneumonia
☐ M35.81, Multisystem inflammatory syndrome associated with COVID-19
☐ N17, acute kidney injury
☐ Z20.822, Contact with and (suspected) exposure to COVID-19
☐ Z20.828, Contact with and (suspected) exposure to other viral communicable diseases
☐ Z86.16, Personal History of COVID-19

Please enter all ICD codes (or, if ICD codes not available, diagnoses) associated with the record not listed above (free text fields)

1. ______________________________
2. ______________________________
3. ______________________________
4. ______________________________
5. ______________________________
6. ______________________________
7. ______________________________
8. ______________________________
9. ______________________________
10. ______________________________

2. Notes (as needed):

3. Reporter: ______________________________

4. Date of report (month / day / year): **Click or tap to enter a date.**

Acute COVID-19 Review Form

[redacted]

Please fill out the following fields based on medical record review. At the end of the form, you will be prompted to complete a final COVID event diagnosis form based on your responses.

- Participant ID: ______________________________

- Event type: select one
  - ☐ Hospitalization
    - Admission date (month / year):_________________________
    - ICU or "step down unit" admission date (if applicable), hospital day (counted from date of admission): _____________________
      Click or tap to enter a date.
    - Discharge date (date of death for deaths), hospital day (counted from day of admission): _____________________
      Click or tap to enter a date.
    - Discharge plan (select one): **Choose an item.**

  - ☐ Death in emergency department
    - Admission date (month / year): _____________________

  - ☐ Out-of-hospital death (month / year): _____________________

- COVID-19 Signs or Symptoms
  - For non-fatal hospitalizations:
    - Were signs or symptoms linked to COVID-19 present at admission?
      Choose an item.
    - If No: Were any signs or symptoms linked to COVID-19 present at any time during the hospital admission?
      Choose an item.

  - For fatal hospitalizations and out-of-hospital deaths:
    - Were signs or symptoms linked to COVID-19 present within four weeks of death?
      Choose an item.
    - Was COVID-19 the cause of death?
      Choose an item.

- COVID-19 test results

  - COVID test performed: ☐ Yes ☐ No
    - If yes:
      - First positive PCR:
        - Hospital day: __________ or ☐N/A or ☐NR

      - First negative PCR
        - Hospital day: __________ or ☐N/A or ☐NR

- First positive antigen test:
    - Hospital day: __________ or ☐N/A or ☐NR

- First positive antibody test:
    - Hospital day: __________ or ☐N/A or ☐NR

- If no positive COVID test, check all that apply:

  ☐ Physician suspicion for COVID-19 (documented as probable/definite diagnosis in discharge summary)

  ☐ Reported exposure to COVID-19

<u>Oxygenation and oxygen supplementation</u>

- **Pre-Admission** information provided ☐ Yes
    - Highest respiratory rate: ___ (min=5, max=50) or ☐N/A or ☐NR
    - Lowest oxygen saturation: ____ (min=0, max=100) or ☐N/A or ☐NR
    - Was this on supplemental oxygen? **Choose an item.**
        - If yes select one:

          ☐ Nasal cannula
          ☐ Face mask
          ☐ High flow
          FiO2: ____% (min=20, max=100) or ☐N/A or ☐NR
          ☐ CPAP
          FiO2: ____% (min=20, max=100) or ☐N/A or ☐NR
          ☐ BiPAP
          FiO2: ____% (min=20, max=100) or ☐N/A or ☐NR
          ☐ Mechanical ventilation
          FiO2: ____% (min=20, max=100) or ☐N/A or ☐NR
          ☐ Not recorded

- Information at **admission**
    - Respiratory rate at **admission**: _____ (min = 5, max = 50) or ☐N/A or ☐NR
    - Oxygen saturation at **admission**: _____ (min=0, max=100) or ☐N/A or ☐NR
    - Was this on supplemental oxygen? **Choose an item.**
        - If yes, select one:

          ☐ Nasal cannula
          ☐ Face mask
          ☐ High flow
          FiO2: ____% (min=20, max=100) or ☐N/A or ☐NR
          ☐ CPAP
          FiO2: ____% (min=20, max=100) or ☐N/A or ☐NR
          ☐ BiPAP
          FiO2: ____% (min=20, max=100) or ☐N/A or ☐NR

☐ Mechanical ventilation
FiO2: ____% (min=20, max=100) or ☐N/A or ☐NR
☐ N/A

- Information over **entire admission**
  - **Lowest** oxygen saturation recorded during **entire hospitalization**: _____ (min=0, max = 100) or ☐ N/A or ☐NR
    ☐ same as admission value

  - Was this on supplemental oxygen? **Choose an item.**
    - If yes, select one:
      ☐ Nasal cannula
      ☐ Face mask
      ☐ High flow
      FiO2: ____% (min=20, max=100) or ☐N/A or ☐NR
      ☐ CPAP
      FiO2: ____% (min=20, max=100) or ☐N/A or ☐NR
      ☐ BiPAP
      FiO2: ____% (min=20, max=100) or ☐N/A or ☐NR
      ☐ Mechanical ventilation
      FiO2: ____% (min=20, max=100) or ☐N/A or ☐NR
      ☐ N/A

  - Supplemental oxygen and respiratory interventions used during **entire hospitalization**, select all that apply:
    ☐ Nasal cannula
    ☐ Face mask
    ☐ High flow
    ☐ CPAP
    ☐ BiPAP
    ☐ Mechanical ventilation
    ☐ Prone position
    ☐ Extra-corporeal membrane oxygenation (ECMO)
    ☐ N/A

- Was an **arterial blood gas (ABG)** performed? ☐ Yes
  If yes:
  - First ABG
    - Hospital day for first ABG: ______ days after admission
    - What was the first ABG result?
      - pH _____ (min 4, max 9)
      - paCO2 ____ mmHg (min 15, max 100)
      - paO2 ____ mmHg (min 5, max 300)
      - Was this on supplemental oxygen? **Choose an item.**
        - If yes, select one:
        ☐ Nasal cannula

- ☐ Face mask
- ☐ High flow
  FiO2: ____% (min=20, max=100) or ☐N/A or ☐NR
- ☐ CPAP
  FiO2: ____% (min=20, max=100) or ☐N/A or ☐NR
- ☐ BiPAP
  FiO2: ____% (min=20, max=100) or ☐N/A or ☐NR
- ☐ Mechanical ventilation
  FiO2: ____% (min=20, max=100) or ☐N/A or ☐NR
- ☐ Extra-corporeal membrane oxygenation (ECMO)

o Was the patient in prone position? ☐ Yes

o What was the ABG with the **lowest paO2** during the **entire hospitalization**?
  - o Same as above ☐ Yes ☐ No

  If no:
  - o Hospital day for ABG: _______ days after admission
  - o pH ______ (min 4, max 9)
  - o paCO2 _____ mmHg (min 15, max 100)
  - o paO2 _____ mmHg (min 5, max 300)
  - o Was this on supplemental oxygen? **Choose an item.**
    - ▪ If yes, select one:
    - ☐ Unknown
    - ☐ No oxygen supplementation (room air)
    - ☐ Nasal cannula
    - ☐ Face mask
    - ☐ High flow
      FiO2: ____% (min=20, max=100) or ☐N/A or ☐NR
    - ☐ CPAP
      FiO2: ____% (min=20, max=100) or ☐N/A or ☐NR
    - ☐ BiPAP
      FiO2: ____% (min=20, max=100) or ☐N/A or ☐NR
    - ☐ Mechanical ventilation
      FiO2: ____% (min=20, max=100) or ☐N/A or ☐NR
    - ☐ Extra-corporeal membrane oxygenation (ECMO)
  - o Was the patient in prone position? ☐ Yes

- Medications used at any point during **entire hospitalization**: check all that apply as Home Rx and/or Hosp Rx
  - ☐ Medications not recorded
  - ☐ Pressors/inotropes
  - ☐ Inhalers
  - ☐ Antibiotics and antivirals
    - ☐ Azithromycin
    - ☐ Chloroquine

- ☐ Favipiravir
- ☐ Hydroxychloroquine
- ☐ Lopinavir/ritonavir
- ☐ Remdesivir
- ☐ Other antibiotics
- ☐ Other Anti-COVID
  If yes: name: ___________

☐ Anti-platelet
- ☐ Aspirin
- ☐ Clopidogrel
- ☐ Dipyridamole
- ☐ Prasugrel
- ☐ Ticagrelor
- ☐ Other Antiplatelet
  If yes: name: _________

☐ Anticoagulant
- ☐ Direct oral anticoagulant (DOAC)
- ☐ Heparin
  - ☐ Subcutaneous? If yes: dose ___ units ___ time(s) per day
  - ☐ Infusion/"drip"?
- ☐ Lovenox/enoxaparin If yes: dose ___ mg/kg ___ time(s) per day
- ☐ Warfarin
- ☐ Other Anticoagulant

☐ Immune modulators
- ☐ Interferons
- ☐ Interleukin-1 inhibitors
- ☐ Interleukin-6 inhibitors
- ☐ Janus kinase inhibitors
- ☐ TNF inhibitors
- ☐ Other Immune modulator If yes: name:________

☐ Antihypertensive
- ☐ Angiotensin converting enzyme (ACE) inhibitors
- ☐ Angiotensin receptor blockers (ARBs)
- ☐ Beta blockers
- ☐ Other Antihypertensive

☐ Medications for hyperglycemia/diabetes
- ☐ Insulin If yes:
  - ☐ Infusion/drip If yes: rate: _____ units/hour
  - ☐ Long-acting (e.g., lantus) If yes: dose: _______ units (min=1, max=150)
  - ☐ Short-acting (e.g., sliding scale meal coverage):
- ☐ Other diabetic medications If yes, name(s): _________

☐ Other Biomedicine If yes: name(s): ____________

☐ NSAIDS

☐ Statins

☐ Steroids
☐ Anti-rejection medicine If yes: name(s): ___________
☐ Other comments (reviewer may list items that are notable/important about medications administered or withheld): _____________

- Pulmonary conditions

☐ Chest X-ray performed:
If yes, was pneumonia present? **Choose an item.**

☐ Chest CT performed:
If yes, was pneumonia present? **Choose an item.**

☐ Discharge diagnosis indicates pneumonia as probable or definite? **Choose an item.**

☐ CT angiogram of the chest performed:
CTA indicative of PE **Choose an item.**
Pneumonia present **Choose an item.**

☐ V/Q scan performed:
V/Q indicative of PE **Choose an item.**

☐ Physician note documenting presumptive PE with initiation of anticoagulation **Choose an item.**

- Cardiac conditions

☐ Troponin tested
  - Highest recorded troponin value (include which troponin test and upper limit of normal for that test):
    - Upper limit of normal for test: _______ (min=0)
    - Highest recorded value: _______ ("alert: above upper limit of normal")

-

☐ Physician documentation of NSTEMI

☐ Physician documentation of STEMI

If YES to physician documentation of NSTEMI or STEMI (or reviewer suspicion for MI):
☐ Acute ischemic changes on ECG
☐ Chest pain

☐ Physician documentation of new atrial fibrillation

If YES to physician documentation of new atrial fibrillation:
☐ Atrial fibrillation on ECG

☐ Echocardiogram performed:

Ejection fraction: _______________ (min=0, max=80)

- Neurological conditions

  ☐ CT head performed:
  CT head consistent with acute/subacute stroke **Choose an item.**

  ☐ MRI brain performed:
  MRI brain consistent with acute/subacute stroke **Choose an item.**

  ☐ Physician documentation of acute/subacute stroke

  ☐ Physician documentation of delirium

- Vascular conditions

  ☐ Lower or upper extremity Doppler/ultrasound performed:
  Extremity Doppler showing new DVT **Choose an item.**
  ☐ Physician documentation of new clot

- Renal conditions

  ☐ Highest creatinine: ______ (min=0, max=12)

  ☐ Lowest creatinine: ______ (min=0, max=12)

  ☐ Baseline creatinine, if documented: ______ (min=0, max=12) or ☐ N/A (-1)

  ☐ Change in creatinine (highest/baseline or, if baseline=N/A, highest/lowest) = CALCULATED, alert: "change in creatinine >1.5, is this correct?" **Choose an item.**

  ☐ Initiation of renal replacement therapy ☐ Yes ☐ On RRT at baseline

  ☐ Physician documentation of acute kidney injury (AKI)

At this time, you may also recommend this record for a second review.

Recommend second review: **Choose an item.**
If yes, please comment: ____

Please confirm that you would like to submit this review form and you will be prompted to complete a final diagnosis form.

Submit

<u>Post-Acute Sequelae of SARS-CoV-2 Infectiton (PASC) Review Form</u>

[redacted]

This form is intended for hospitalization for post-acute sequelae of SARS-CoV-2 infection (PASC; infection confirmed 30+ days prior to admission).

Please fill out the following fields based on medical record review. At the end of the form, you will be prompted to complete a final COVID event diagnosis form based on your responses.

- Participant ID: ___________________________

☐ Admission date (month / year):_______________________
- o ICU admission date (if applicable), hospital day (counted from date of admission): ___________________
  Click or tap to enter a date.
- o "Step down unit" admission date (if applicable), hospital day (counted from date of admission): ___________________
  Click or tap to enter a date.
- o Discharge date (date of death for deaths), hospital day (counted from day of admission): ___________________
  Click or tap to enter a date.
- o Discharge plan (select one): **Choose an item.**

What was the established date of the first COVID-19 infection? (Date: _________)

Was the patient previously admitted for acute SARS-CoV-2 Infection? ☐ Yes ☐ No

If yes:
- What was the length of Stay?
- How long was the time from discharge to this admission?
- Did the patient have any of the following treatments during the prior admission?

  ☐Steroids
  ☐Remdesivir
  ☐Paxlovid
  ☐monoclonal antibodies
  ☐plasma
  ☐HFNC
  ☐intubation
  ☐Bi-level
  ☐ICU admission

During the prior admission(s) for acute COVID, were any of the following conditions reported?

☐Stroke

☐Heart attack
☐DVT/PE
☐Renal failure
☐Pneumonia
☐Other:_________

- COVID-19 test results for this admission

  - COVID PCR performed: ☐ Yes ☐ No
  - If yes: COVID PCR positive? Yes/No

What symptoms has the patient experienced since their prior SARS-CoV-2 admission? (Check all that apply)

☐Fatigue
☐Weakness
☐Dizziness
☐Joint or muscle pain

☐Headache
☐Cough
☐Shortness of breath
☐Loss of taste or smell
☐Chest pain
☐Delirium
☐Sleep disturbance
☐Diarrhea
☐Nausea or vomiting
☐Memory or concentration problems
☐Other: ________

Oxygenation and oxygen supplementation

- Information at **admission**
  - Respiratory rate at **admission**: ______ (min = 5, max = 50) or ☐N/A or ☐NR
  - Oxygen saturation at **admission**: ______ (min=0, max=100) or ☐N/A or ☐NR
  - Was this on supplemental oxygen? **Choose an item.**
    - If yes, select one:

      ☐ Nasal cannula
      ☐ Face mask
      ☐ High flow
      FiO2: ____% (min=20, max=100) or ☐N/A or ☐NR
      ☐ CPAP
      FiO2: ____% (min=20, max=100) or ☐N/A or ☐NR

☐ BiPAP
FiO2: _____% (min=20, max=100) or ☐N/A or ☐NR
☐ Mechanical ventilation
FiO2: _____% (min=20, max=100) or ☐N/A or ☐NR
☐ N/A

- Supplemental oxygen and respiratory interventions used during **entire hospitalization**, select all that apply:
  ☐ Nasal cannula
  ☐ Face mask
  ☐ High flow
  ☐ CPAP
  ☐ BiPAP
  ☐ Mechanical ventilation
  ☐ Prone position
  ☐ Extra-corporeal membrane oxygenation (ECMO)
  ☐ N/A

- Pulmonary conditions

☐ Chest X-ray performed:
If yes, was pneumonia present? **Choose an item.**
If yes, were fibrotic changes present? YES/NO

☐ Chest CT performed:
If yes, was pneumonia present? **Choose an item.**
If yes, were fibrotic changes present? YES/NO

☐ Discharge diagnosis indicates pneumonia as probable or definite? **Choose an item.**
Discharge diagnosis indicates pulmonary fibrosis as probable or definite?
☐ CT angiogram of the chest performed:
CTA indicative of PE **Choose an item.**
Pneumonia present **Choose an item.**
If yes, were fibrotic changes present? YES/NO
☐ V/Q scan performed:
V/Q indicative of PE **Choose an item.**

☐ Physician note documenting presumptive PE with initiation of anticoagulation **Choose an item.**

- Cardiac conditions (NOTE: THE FOLLOWING ITEMS ARE IDENTICAL TO THE ORIGINAL EVENTS REVIEW ITEMS)

☐ Troponin tested

- Highest recorded troponin value (include which troponin test and upper limit of normal for that test):
    - Troponin name:____________________
    - Upper limit of normal for test: _______ (min=0)
    - Highest recorded value: ______ ("alert: above upper limit of normal")

- ☐ Physician documentation of NSTEMI **Choose an item.**

  ☐ Physician documentation of STEMI **Choose an item.**

  ☐ ST Elevation ECG **Choose an item.**

  ☐ Chest pain or equivalent

  ☐ Echocardiogram performed:
  Ejection fraction: _______________ (min=0, max=80)

  ☐ Was there a new diagnosis of atrial fibrillation? **Choose an item.**

- Neurological conditions

  ☐ CT head performed:
  CT head consistent with acute/subacute stroke **Choose an item.**

  ☐ MRI brain performed:
  MRI brain consistent with acute/subacute stroke **Choose an item.**

  ☐ Physician documentation of acute/subacute stroke

  ☐ Physician documentation of delirium

- Vascular conditions

  ☐ Lower or upper extremity Doppler/ultrasound performed:
  Extremity Doppler showing new DVT **Choose an item.**
  ☐ Physician documentation of new clot

- Renal conditions

  ☐ Highest creatinine: _____ (min=0, max=12)

  ☐ Lowest creatinine: _____ (min=0, max=12)

  ☐ Baseline creatinine, if documented: _____ (min=0, max=12) or ☐ N/A

☐ Change in creatinine (highest/baseline or, if baseline=N/A, highest/lowest) = CALCULATED, alert: "change in creatinine >1.5, is this correct?" **Choose an item.**

☐ Initiation of renal replacement therapy (RRT) ☐ Yes ☐ On RRT at baseline

☐ Physician documentation of acute kidney injury (AKI)

At this time, you may also recommend this record for a second review.

Recommend second review: **Choose an item.**
If yes, please comment: ____

Please confirm that you would like to submit this review form and you will be prompted to complete a final diagnosis form.

Submit

<u>Diagnosis Form:</u> If Event Type = Hospitalization or deceased in emergency department

[redacted]

Thank you for completing the COVID event review form. Please review the diagnoses supported by the information from your review, combined with ICD codes for the event, and then provide your diagnoses.
Please note that if you choose to differ from the supported diagnoses, this record will be referred for a second review. You may provide any additional comments at the end of the form.

COVID-19 INFECTION

Probable criteria	Met?	Definite criteria	Met?
Physician suspicion	☐ Yes	U07.1	☐ Yes
Informant suspicion	☐ Yes	U09.9	☐ Yes
Acute COVID-like illness (fever, cough, and/or shortness of breath without other identifiable cause) without positive COVID-19 test	☐ Yes	M35.81	
		J12.82	☐ Yes
		Positive PCR	☐ Yes
		Positive antigen test	☐ Yes

Reviewer diagnosis (select one): **Choose an item.**

COVID-19 HOSPITALIZATION

Probable criteria	Met?	Definite criteria	Met?
Probable COVID-19 infection*	☐ Yes	Definite COVID-19 infection*	☐ Yes
Admitted to hospital for COVID-related signs or symptoms or developed COVID-related signs or symptoms during hospitalization	☐ Yes	Admitted to hospital for COVID-related signs or symptoms or developed COVID-related signs or symptoms during hospitalization	☐ Yes
Deceased in ED	☐ Yes	Deceased in ED	☐ Yes

*Required; needs at least one additional criterion

Reviewer diagnosis (select one): **Choose an item.**

COVID-19 SEVERE ILLNESS

Please note that this diagnosis is not required for PASC events.

Probable criteria	Met?	Definite criteria	Met?
Probable COVID-19 infection*	☐ Yes	Definite COVID-19 infection*	☐ Yes
Oxygen saturation < 94% (on room air or on O2 supplementation) at any time, including pre-admission	☐ Yes	Oxygen saturation < 94% (on room air or on O2 supplementation) at any time, including pre-admission	☐ Yes
Respiratory rate > 30 breaths/minute	☐ Yes	Respiratory rate > 30 breaths/minute	☐ Yes
Required BiPAP, HFNC, MV, and/or ECMO	☐ Yes	Required BiPAP, HFNC, MV, and/or ECMO	☐ Yes

*Required; needs at least one additional criterion

Reviewer diagnosis (select one): **Choose an item.**

COVID-19 CRITICAL ILLNESS

Please note that this diagnosis is not required for PASC events.

Probable criteria	Met?	Definite criteria	Met?
Probable COVID-19 infection*	☐ Yes	Definite COVID-19 infection*	☐ Yes
High flow oxygen	☐ Yes	High flow oxygen	☐ Yes
Mechanical ventilation	☐ Yes	Mechanical ventilation	☐ Yes
ECMO	☐ Yes	ECMO	☐ Yes
Inotropes/pressors	☐ Yes	Inotropes/pressors	☐ Yes
ICU admission	☐ Yes		
Step down unit admission	☐ Yes		

*Required; needs at least one additional criterion

Reviewer diagnosis (select one): **Choose an item.**

COVID-19 PNEUMONIA

Probable criteria	Met?	Definite criteria	Met?
Probable COVID-19 infection*	☐ Yes	Definite COVID-19 infection*	☐ Yes
J12.82	☐ Yes	Chest CT with pneumonia	☐ Yes
Physician documentation of pneumonia	☐ Yes	Chest X-ray with pneumonia	☐ Yes
J12.89	☐ Yes		

*Required; needs at least one additional criterion

Reviewer diagnosis (select one): **Choose an item.**

COVID-19 MYOCARDIAL INFARCTION

Probable criteria	Met?	Definite criteria	Met?
Probable COVID-19 infection*	☐ Yes	Definite COVID-19 infection*	☐ Yes
I21	☐ Yes	Positive troponin*	☐ Yes
Physician documentation of STEMI	☐ Yes	Acute ischemic changes on ECG	☐ Yes
Physician documentation of NSTEMI	☐ Yes	Chest pain or equivalent	☐ Yes

*Required; needs at least one additional criterion

Reviewer diagnosis (select one): **Choose an item.**

COVID-19 STROKE

Probable criteria	Met?	Definite criteria	Met?
Probable COVID-19 infection*	☐ Yes	Definite COVID-19 infection*	☐ Yes
ICD-10 I63	☐ Yes	CT head consistent with acute/subacute stroke	☐ Yes
Physician documentation of acute/subacute stroke	☐ Yes	MRI brain consistent with acute/subacute stroke	☐ Yes

Reviewer diagnosis (select one): **Choose an item.**

COVID-19 PULMONARY EMBOLISM

Probable criteria	Met?	Definite criteria	Met
Probable COVID-19 infection*	☐ Yes	Definite COVID-19 infection*	☐ Yes
I26	☐ Yes	CTA indicative of PE	☐ Yes
Physician note documenting presumptive PE with initiation of anticoagulation	☐ Yes	VQ indicative of PE	☐ Yes

*Required; needs at least one additional criterion

Reviewer diagnosis (select one): **Choose an item.**

COVID-19 DVT

Probable criteria	Met?	Definite criteria	Met?
Probable COVID-19 infection*	☐ Yes	Definite COVID-19 infection*	☐ Yes
I82	☐ Yes	Lower extremity Doppler showing new/active DVT	☐ Yes
Physician documentation of new/active clot	☐ Yes		☐ Yes

*Required; needs at least one additional criterion

Reviewer diagnosis (select one): **Choose an item.**

COVID-19 RENAL FAILURE

Probable criteria	Met?	Definite criteria	Met?
Probable COVID-19 infection*	☐ Yes	Definite COVID-19 infection*	☐ Yes
N17	☐ Yes	Max/min or Max/baseline creatinine > 1.5	☐ Yes
Physician documentation of acute kidney injury (AKI)	☐ Yes	New initiation of renal replacement therapy	☐ Yes

*Required; needs at least one additional criterion

Reviewer diagnosis (select one): **Choose an item.**

FATAL COVID-19

Probable criteria	Met?	Definite criteria	Met?
Death*	☐ Yes	Death*	☐ Yes
Probable COVID-19 hospitalization*	☐ Yes	Definite COVID-19 hospitalization*	☐ Yes
Judged to be probable cause of death	☐ Yes	Judged to be definite cause of death	☐ Yes

*Required

Reviewer diagnosis (select one): **Choose an item.**

FOR ALL EVENT TYPES

Comments: ____

Would you like to refer this record for a second review?

☐ Yes
☐ No

<u>Diagnosis Form:</u> If Event Type = Out of Hospital Death

[redacted]

COVID-19 INFECTION

Probable criteria	Met? (Y/N)	Definite criteria	Met (Y/N)
Physician suspicion		U07.1	
Informant suspicion		U09.9	
Acute COVID-like illness (fever, cough, and/or shortness of breath without other identifiable cause) without positive COVID-19 test		M35.81	
		J12.82	

Reviewer diagnosis (select one): **Choose an item.**

FATAL COVID-19

Probable criteria	Met? (Y/N)	Definite criteria	Met (Y/N)
Physician suspicion		U07.1	
Informant suspicion		U09.9	
Acute COVID-like illness (fever, cough, and/or shortness of breath without other identifiable cause) without positive COVID-19 test within 28 days of death		M35.81	
Judged to be probable cause of death		J12.82	
		Judged to be definite cause of death	

Reviewer diagnosis (select one): **Choose an item.**

FOR ALL EVENT TYPES

Comments: ____

Would you like to refer this record for a second review?

☐ Yes
☐ No
