# Supplementary material for: Classifying COVID-19 hospitalizations in epidemiology cohort studies: The C4R study
Source: PLoS One. 2025 Feb 10;20(2):e0316198. doi: 10.1371/journal.pone.0316198 (PMC11809881; doi:10.1371/journal.pone.0316198)
Supplement: S1 Table — (DOCX) [file pone.0316198.s003.docx]

**S1 Table. Characteristics of participants in C4R cohorts, United States, March 1, 2020.**

| Cohort | N | Original enrollment | Current age range, years | Sex, % | Race/ethnicity, % | | | | | | Original research focus |
| --- | --- | --- | --- | --- | --- | --- | --- | --- | --- | --- | --- |
|  |  |  |  | Female | NHW | B | H/L | As | Am Ind | Other |  |
| ARIC | 6,690 | 1987-89 | 75-97 | 63 | 77 | 23 | 0^a^ | 0 | 0 | 0 | Cardiovascular |
| CARDIA | 4,590 | 1985-86 | 53-66 | 56 | 50 | 50 | 0 | 0 | 0 | 0 | Cardiovascular |
| COPDGene | 7,731 | 2007-12 | 50-90 | 48 | 65 | 35 | 0 | 0 | 0 | 0 | Pulmonary |
| FHS | 7,339 | 1971-2005 | 26-108 | 56 | 86 | 3 | 4 | 0 | 0 | 7 | Cardiovascular |
| HCHS/SOL | 13,142 | 2008-11 | 30-87 | 60 | 0 | 0 | 100 | 0 | 0 | 0 | Cardiovascular |
| JHS | 2,444 | 2000-04 | 38-102 | 63 | 0 | 100 | 0 | 0 | 0 | 0 | Cardiovascular |
| MASALA | 1,132 | 2010-13 | 50-94 | 47 | 0 | 0 | 0 | 100 | 0 | 0 | Cardiovascular |
| MESA | 4,683 | 2000-02 | 65-103 | 56 | 38 | 27 | 24 | 12 | 0 | 0 | Cardiovascular |
| NOMAS | 1,256 | 1993-2003 | 62-106 | 65 | 12 | 14 | 72 | 0 | 1 | 0 | Neurologic |
| PrePF | 5,000 | 2000-13 | 40-80 | 55 | 92 | 3 | 3 | 0 | 0 | 0 | Pulmonary |
| REGARDS | 12,766 | 2003-07 | 57-105 | 58 | 62 | 38 | 0 | 0 | 0 | 0 | Neurologic |
| SARP | 397 | 2000-present | 18-80 | 65 | 75 | 25 | 0 | 0 | 0 | 0 | Pulmonary |
| SPIROMICS | 2,273 | 2010-15 | 47-87 | 48 | 82 | 4 | 4 | 0 | 0 | 0 | Pulmonary |
| SHS | 2,915 | 1984-94 | 31-105 | 62 | 0 | 0 | 0 | 0 | 100 | 0 | Cardiovascular |

Am Ind = American Indian; As = Asian American; B = Black; H/L = Hispanic/Latinx; NHW = Non-Hispanic White.

ARIC = Atherosclerosis Risk in Communities Study; C4R = Collaborative Cohort of Cohorts for COVID-19 Research; CARDIA = Coronary Artery Risk Development in Young Adults; COPDGene= Genetic Epidemiology of COPD; FHS = Framingham Heart Study; HCHS/SOL = Hispanic Community Health Study/Study of Latinos; JHS = Jackson Heart Study; MASALA = Mediators of Atherosclerosis in South Asians Living in America; MESA = Multi-Ethnic Study of Atherosclerosis; NOMAS = Northern Manhattan Study; PrePF = Prevent Pulmonary Fibrosis; REGARDS = REeasons for Geographic and Racial Differences in Stroke; SARP = Severe Asthma Research Program; SPIROMICS = Subpopulations and Intermediate Outcome Measures in COPD Study; SHS = Strong Heart Study

^a^ ARIC did not inquire regarding Hispanic/Latino ethnicity, hence White participants cannot be definitely defined as non-Hispanic.
