# Supplementary material for: Classifying COVID-19 hospitalizations in epidemiology cohort studies: The C4R study
Source: PLoS One. 2025 Feb 10;20(2):e0316198. doi: 10.1371/journal.pone.0316198 (PMC11809881; doi:10.1371/journal.pone.0316198)
Supplement: S3 Table — (DOCX) [file pone.0316198.s005.docx]

**S3 Table. Selected COVID-19 questionnaire elements and their inclusion by cohorts in questionnaires for C4R, United States, April 2020 – February 2023.**

| Question | N, Cohorts |
| --- | --- |
| Do you think that you have had COVID-19? | 14 |
| Did a healthcare provider ever tell you that you had COVID-19? | 13 ^a^ |
| Have you ever had a test that showed you had COVID-19? | 14 |
| When you knew or thought that you had COVID-19 the first time, did you have any symptoms? | 14 |
| Since March 1, 2020, have you had an overnight stay in a hospital for any illness related to COVID-19? | 14 |
| While in the hospital, did you have any of the following treatments: A breathing tube or ventilator? ICU monitoring? | 10 ^b^ |

^a^Not included in Wave 1 Questionnaire in REGARDS.

^b^Not included in Wave 1 Questionnaire in ARIC, CARDIA, HCHS/SOL, or REGARDS.
