# Supplementary material for: Classifying COVID-19 hospitalizations in epidemiology cohort studies: The C4R study
Source: PLoS One. 2025 Feb 10;20(2):e0316198. doi: 10.1371/journal.pone.0316198 (PMC11809881; doi:10.1371/journal.pone.0316198)
Supplement: S4 Table — (DOCX) [file pone.0316198.s006.docx]

**S4 Table. Criteria for classification of COVID-19 diagnoses as definite or probable based on medical record review.**

| Classification | Probable | Definite |
| --- | --- | --- |
| SARS-CoV-2 Infection | Any of the following:   - Physician suspicion - Informant suspicion - Acute COVID-19-like illness (fever, cough, and/or shortness of breath without other identifiable cause) | Any of the following   - ICD code for SARS-CoV-2 infection or related illness (U07.1, U09.9, M35.81, J12.82) - Positive PCR or antigen test in medical record |
| COVID-19 illness severity | | |
| *COVID-19 Hospitalization* | Probable infection AND any of the following:   - Admitted to the hospital for COVID-19-related signs or symptoms; or, developed COVID-19-related signs or symptoms during hospitalization - Deceased in Emergency Department | Definite infection AND any of the following:   - Admitted to the hospital for COVID-19-related signs or symptoms; or, developed COVID-19-related signs or symptoms during hospitalization - Deceased in Emergency Department |
| *COVID-19 Severe Illness* | Probable infection AND any of the following:   - Oxygen saturation < 94% (on room air or on O2 supplementation) at any time, including pre-admission - Respiratory rate > 30 breaths/minute - Required BiPAP, HFNC, MV, and/or ECMO | Definite infection AND any of the following:   - Oxygen saturation < 94% (on room air or on O2 supplementation) at any time, including pre-admission - Respiratory rate > 30 breaths/minute - Required BiPAP, HFNC, MV, and/or ECMO |
| *COVID-19 Critical Illness* | Probable infection AND any of the following:   - High-flow oxygen - Mechanical ventilation or ECMO - Inotropes or pressors - ICU or step down unit admission | Definite infection AND any of the following:   - High-flow oxygen - Mechanical ventilation or ECMO - Inotropes or pressors |
| *Fatal COVID-19* | Probable infection AND:   - Probable COVID-19 hospitalization ending in death, *OR* - Physician or other informant suspicion of COVID-19 contributing to death, *OR* - Acute COVID-19-like illness (fever, cough, and/or shortness of breath without other identifiable cause) without positive COVID-19 test within 28 days of death, *OR* - Judged by reviewer that COVID-19 is probable cause of death | Definite infection AND:   - Death certificate with ICD:   - U07.1, Confirmed COVID-19   - U09.9, Post-infectious state after COVID-19   - M35.81, Multisystem inflammatory syndrome associated with COVID-19   - J12.82, Pneumonia due to coronavirus disease 2019, *OR* - Definite COVID-19 hospitalization ending in death, *OR* - Judged by reviewer that COVID-19 is definite cause of death |
| COVID-19 complications | | |
| *COVID-19 Pneumonia* | Probable infection AND any of the following:   - ICD code for COVID-19 pneumonia (J12.82, J12.89) - Physician documentation of pneumonia | Definite infection AND any of the following:   - Chest CT with pneumonia - Chest X ray with pneumonia |
| *COVID-19 MI* | Probable infection AND:   - ICD-10 I21, *OR* - Physician documentation of NSTEMI, *OR* - Physician documentation of STEMI | Definite infection AND:   - ST Elevation on electrocardiogram consistent with STEMI and troponin greater than the upper limit of normal (if available), *or* - Pathologic evidence of acute myocardial infarction |
| *COVID-19 Stroke* | Probable infection AND:   - ICD-10 I63, *OR* - Physician documentation of stroke | Definite infection AND:   - CT head consistent with stroke, *OR* - MRI brain consistent with stroke |
| *COVID-19 PE* | Probable infection AND:   - ICD-10 I26, *OR* - Physician note documenting presumptive PE with initiation of anticoagulation | Definite infection AND:   - CTA indicative of PE, *OR* - V/Q indicative of PE |
| *COVID-19 DVT* | Probable infection AND:   - ICD-10 I82, *OR* - Physician note documenting new venous thrombosis | Definite infection AND:   - Lower extremity Doppler showing DVT |
| *COVID-19 Renal Failure* | Probable infection AND:   - ICD-10 N17, *OR* - Physician documentation of acute kidney injury | Definite infection AND:   - Max/min or Max/baseline creatinine > 1.5, *OR* - New initiation of renal replacement therapy |

BiPAP = bilevel positive airway pressure ventilator; COVID-19 = coronavirus disease 2019; CT = computed tomography; ECMO = extracorporeal membrane oxygenation; HFNC = high-flow nasal canula; ICD = international classification of diseases; MV = mechanical ventilation; NSTEMI = non-ST elevation myocardial infarction; PCR = polymerase chain reaction; PE = pulmonary embolism; STEMI = ST elevation myocardial infarction.
